# A genetic screen in *C*. *elegans* reveals roles for KIN17 and PRCC in maintaining 5’ splice site identity

**DOI:** 10.1371/journal.pgen.1010028

**Published:** 2022-02-10

**Authors:** Jessie M. N. G. L. Suzuki, Kenneth Osterhoudt, Catiana H. Cartwright-Acar, Destiny R. Gomez, Sol Katzman, Alan M. Zahler

**Affiliations:** 1 Center for Molecular Biology of RNA, Department of Molecular Cell Developmental Biology, University of California, Santa Cruz, California, United States of America; 2 UCSC Genomics Institute, University of California, Santa Cruz, Santa Cruz, California, United States of America; Ohio State University, UNITED STATES

## Abstract

Pre-mRNA splicing is an essential step of eukaryotic gene expression carried out by a series of dynamic macromolecular protein/RNA complexes, known collectively and individually as the spliceosome. This series of spliceosomal complexes define, assemble on, and catalyze the removal of introns. Molecular model snapshots of intermediates in the process have been created from cryo-EM data, however, many aspects of the dynamic changes that occur in the spliceosome are not fully understood. *Caenorhabditis elegans* follow the GU-AG rule of splicing, with almost all introns beginning with 5’ GU and ending with 3’ AG. These splice sites are identified early in the splicing cycle, but as the cycle progresses and “custody” of the pre-mRNA splice sites is passed from factor to factor as the catalytic site is built, the mechanism by which splice site identity is maintained or re-established through these dynamic changes is unclear. We performed a genetic screen in *C*. *elegans* for factors that are capable of changing 5’ splice site choice. We report that KIN17 and PRCC are involved in splice site choice, the first functional splicing role proposed for either of these proteins. Previously identified suppressors of cryptic 5’ splicing promote distal cryptic GU splice sites, however, mutations in KIN17 and PRCC instead promote usage of an unusual proximal 5’ splice site which defines an intron beginning with UU, separated by 1nt from a GU donor. We performed high-throughput mRNA sequencing analysis and found that mutations in PRCC, and to a lesser extent KIN17, changed alternative 5’ splice site usage at native sites genome-wide, often promoting usage of nearby non-consensus sites. Our work has uncovered both fine and coarse mechanisms by which the spliceosome maintains splice site identity during the complex assembly process.

## Author summary

Pre-messenger RNA splicing is an important regulator of eukaryotic gene expression, changing the content, frame, and functionality of both coding and non-coding transcripts. Our understanding of how the spliceosome chooses where to cut has focused on the initial identification of splice sites. However, our results suggest that the spliceosome also relies on other components in later steps to maintain the identity of the splice donor sites. We are currently in the midst of a “resolution revolution”, with ever-clearer cryo-EM snapshots of stalled complexes, allowing researchers to visualize moments in time in the splicing cycle. These models are illuminating, but do not always elucidate mechanistic functioning of a highly dynamic ribonucleoprotein complex. Therefore, our lab takes a complementary approach, using the power of genetics in a multicellular animal to gain functional insights into the spliceosome. Using a *C*.*elegans* genetic screen, we have found novel functional splicing roles for two proteins, KIN17 and PRCC. Mutations in PRCC in particular promote nearby alternative 5’ splice sites at native loci. This work improves our understanding of how the spliceosome maintains the identity of where to cut the pre-mRNA, and thus how genes are expressed and used in multicellular animals.

## Introduction

The spliceosome is not one distinct machine but a series of dynamic macromolecular protein/RNA complexes that assemble on and catalyze the removal of introns from pre-mRNA transcripts in eukaryotic organisms. Over one hundred proteins, including multiple helicases, and the 5 U-rich small nuclear RNAs (snRNAs) join, rearrange, and withdraw from a spliceosomal complex in a choreographed sequence over the course of a single splicing cycle, catalyzing the removal of an intron and ligation of the flanking exons [1,2]. Spliceosomes assemble *de novo* from subunits on each nascent pre-mRNA intron. Multiple spliceosomes often interact with a pre-mRNA transcript at the same time, and different introns in a pre-mRNA can have different kinetics for removal [3]. The splicing process is responsible for an essential information processing step in the flow of genetic information, and almost all protein-coding transcripts in metazoans must be spliced in order to become functional.

Mutations in splice sites or in cis-regulatory regions, such as enhancer or silencer binding sites, can cause a variety of deleterious splicing phenotypes that are associated with disease phenotypes. Examples include exon skipping, intron inclusion, and frameshifts. In addition to alteration of regulatory elements, mutation of a splicing donor or acceptor sequence can lead to activation of nearby “cryptic” splice sites, which are defined as splice sites that are functional but activated only when an authentic splice site is disrupted by mutation. In the Human Gene Mutation Database, ~9% of inherited disease-causing mutations alter splice site sequences [4], and another ~25% of disease-causing mutations affect splicing by disrupting other important sequences, such as nearby regulatory binding sites [5,6]. Some aberrant mRNAs are degraded by non-stop, or nonsense-mediated decay pathways, so that the possibly toxic effects of aberrant mRNAs are not amplified into many aberrant proteins by polyribosomes [7]. Precise splicing is central to gene expression, and mutations that affect splicing can lead to a variety of deleterious phenotypes.

Early in the metazoan splicing cycle, three important landmarks on the nascent pre-mRNA are identified by spliceosomal components: the 5’ splice site (exon/intron boundary), the branchpoint, and the 3’ splice site (intron/exon boundary). The U1 snRNA has a 9 base sequence, 3’ GUCCAψψCAUA 5’ that pairs with the bases of the 5’ splice site [8]. A perfectly complementary 5’ splice site would have the sequence 5’ CAG/GUAAGUAU 3’, where the slash represents the splice site, however, this exact sequence is rarely found at verified 5’ splice sites in metazoans. Instead, a consensus sequence that has some overall base-pairing ability with U1snRNA, with a strong preference for a /GU dinucleotide to start the intron, is seen [9]. The 5’ phosphate of the /G will link directly to the branchpoint adenosine. For the 3’ss, the U2AF heterodimer initially identifies the polypyrimidine tract and AG dinucleotide at the end of the intron; U2AF65 binds the polypyrimidine tract, and U2AF35 binds the nearly invariant AG/ at the very 3’ end of the intron [10]. U2AF helps to recruit U2 snRNP to the branch site where base-pairing interactions with U2snRNA, in which the branch point adenosine is bulged out of the duplexed region, define the branchpoint [10,11].

Throughout the many dynamic assembly steps of the splicing cycle, the U1-identified 5’ splice site is maintained by a series of protein and snRNA escorts. In the earliest steps of spliceosome assembly, the 5’ splice site is directly bound by U1 snRNA [12]. In the transition from pre-B to B-complex, U1 leaves the spliceosome while the 5’ splice site is handed off to U6 and residues of PRP8 [13,14]. From B complex, the spliceosome undergoes a number of rearrangements through pre-B^act^1, pre-B^act^2, B^act^, and C complex. CryoEM studies of these complexes from human spliceosomes [2,15] allow for the study of different snapshots of the spliceosome assembly process. In these complexes, there is an exchange of different factors that interact with the region of the 5’ss, as well as with the U6 ACAGAGA box, as the 5’ss is loaded into the catalytic core of the splicing machine. Proteins and snRNPs that bind to the 5’ splice site must bind precisely to a degenerate sequence on a long nucleotide chain, maintain their exact binding position through helicase-powered translocations and substantial conformational changes, and then transfer custody of the 5’ splice site to the next escort without introducing positional error. It is still unclear which components of the spliceosome ensure that the handoffs between escorts will not result in small shifts in 5’ splice site definition.

Thanks to the researchers fueling the ongoing cryo-EM resolution revolution, we now have structures of spliceosomes at many time points in the splicing cycle. These snapshots of experimentally stalled spliceosome assemblies offer valuable insights into the complex assembly pathways, rearrangements, and interactions of spliceosomal components [2]. Mass spectrometry experiments and chemical probing of structures have provided additional information about where and when specific components are associated with the spliceosome during the splicing cycle. These advances continue to build towards a fuller picture of the many multi-step assembly pathways of the splicing cycle and the organized dissolution of the complex. While the structuralists reveal which proteins are where, geneticists are positioned to provide complementary insights into the functional roles of splicing components in splice site choice.

**Fig 1:**
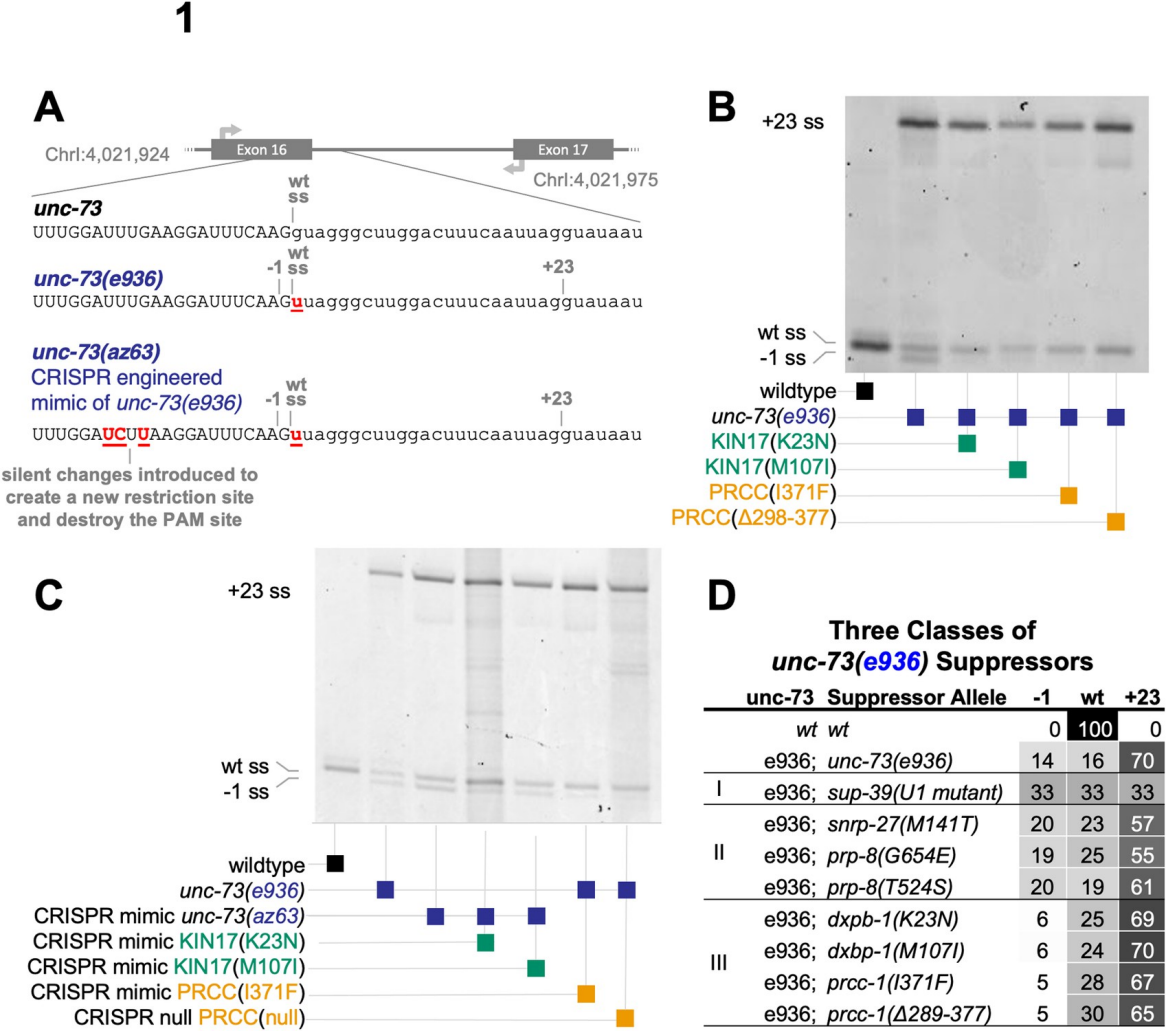
Mutations in KIN17/dxbp-1 and PRCC suppress cryptic splicing, promoting an unusual /UU 5’ splice site. **(A)** Schematic diagram of the 16th intron of the *C*. *elegans* gene *unc-73*, showing genomic coordinates and relative loci of splice sites and PCR primer locations used to assess splice site usage. Below, aligned sequences of the *unc-73* sequence and exon/intron boundary in wild type, *unc-73(e936)*, and in the CRISPR engineered allele *unc-73(az63)*. The cryptic splice sites activated in the competition assay are labeled -1 and +23 and define introns beginning with /GU that are both out of frame. Note that the wild-type splicing position is still denoted “wt ss” even though that intron now begins /UU. The slash mark (/) denotes the splice site. **(B)** Poly-acrylamide gel showing Cy-3 labeled *unc-73* PCR products amplified from *unc-73* cDNA. RNA was extracted from plates of the following 6 mixed-stage strains of *C*. *elegans*: wild-type N2, *unc-73(e936)*, and four independent original suppressed strains identified in the genetic screen whose genotypes are indicated below, each bears both the unc-73(e936) allele and an extragenic suppressor of both the movement defect. The same PCR primers are used on all samples; band positions and intensities are indicative of relative use of the three available 5’ splice sites, labeled -1, wt, and +23. Strains are, in lane order, N2, SZ181, SZ162, SZ283, SZ280, and SZ281, see Methods for genetic details. **(C)** Putative suppressor identities were verified by *de novo* recreations of mutations using CRISPR/Cas9 and homology-directed repair into *unc-73* reporter strains. Image is a scan of a denaturing polyacrylamide gel showing Cy-3 labeled *unc-73* PCR products from *unc-73* cDNA. RNA was extracted from mixed-stage strains with the indicated *unc-73*, *dxbp-1*, and *prcc-1* alleles shown below. Strains are, in lane order, N2, SZ181, SZ219, SZ391, SZ222, SZ308, SZ348, see Methods for genetic details. Unless otherwise mentioned, CRISPR-engineered mimic alleles are used for all subsequent experiments and figures in this report. **(D)** Four new suppressors of cryptic splicing represent a new class of suppressors, with a distinct molecular phenotype compared to previously identified suppressors. Indicated are the suppressor class (I, II, or III) [16,17,20], genotype of *unc-73*, genotype of suppressor, average percent spliced in (PSI), n≥3, at the /GU splice site at position -1 relative to wild type, average PSI at /UU splice site in wild-type position, and average PSI at the /GU at position +23. Conditional grayscale shading highlights patterns in numerical data. All 4 Type III suppressors have a statistically significant difference in usage of the -1 splice site when compared to all of the Type II suppressors, p< 0.01 by Student’s T-Test. (S1 Table). Note that the values in 1D for Type II suppressors and control vary slightly from previous publications, but the trends are all consistent. This variation may be due to the use of the new Cy-3-labeled primer assay, see Methods.

Our lab has previously made use of an unusual 5’ splice site mutation in *C*. *elegans* as a tool to reveal residues on splicing proteins that can contribute to splice site choice [16,17]. UNC-73 is a guanine nucleotide exchange factor that is important in axon guidance and other aspects of *C*. *elegans* development. A fortuitous G->U mutation of the first nucleotide of the 16th intron of the *unc-73* gene, allele *e936* (ce10::chrI:4,021,954) [18] converts the nearly invariant /GU dinucleotide found at the beginning of eukaryotic introns to a /UU dinucleotide, creating a curiously ambiguous splice site (Fig 1A). This splice site mutation results in missplicing, causing the uncoordinated (unc) phenotype [19]. This dramatic phenotype is corrected by even a small increase in in-frame splicing, making its suppression screenable. Previously identified dominant mutations that can suppress the unc phenotype by altering cryptic splicing in *unc-73(e936)* were found in U1snRNA [20], SNRP-27 [16, 21] and the largest and most conserved protein in the spliceosome, PRP-8 [17]. The suppressive role these mutations play in this splice site assay provided genetic evidence of a role for these protein residues in 5’ splice site choice. After publishing these data, the progress made in cryo-EM and crystal structures of the spliceosome has allowed these suppressor alleles to be precisely mapped in the high-resolution inner core of spliceosomal structures; these mutations are often modeled near the active site of the spliceosome providing some clues as to mechanisms for maintaining the identity of the 5’ss during spliceosome assembly. There has been incredible progress in spliceosomal structure studies through cryo-EM, but it has been argued for complementary genetic and biochemical approaches to understand spliceosome mechanism [22].

Here we report new additional suppressor alleles identified in the *unc-73(e936)* genetic screen for suppression of uncoordination that have a dramatically different mechanism of suppression through splicing. Previous suppressors promoted the use of both the -1 and wt cryptic sites separated by 1nt, /G/UU, over a downstream cryptic GU splice donor at position +23. Here we identify two new proteins as splicing factors in which mutations promote use of the /UU splice donor over the adjacent GU splice site. Two missense alleles in the worm homolog of **KIN17** (Kinship to RecA), called *dxbp-1* (downstream of x-box protein) in *C*. *elegans*, and an overlapping point mutation and deletion in the worm homolog of human **PRCC** (proline-rich coiled coil protein or papillary renal cell carcinoma protein), called *prcc-1* in *C*. *elegans*, promote the usage of an unusual /UU splice site in 3-choice, 2-choice and 2X2-choice cryptic splice site assays. High throughput mRNA-SEQ studies reveal that these mutations affect global splicing at native splice sites, but despite similarities in effects on *unc-73(e936)* cryptic splicing, mutations in KIN17 and PRCC display different effects on native genes. These results are the first demonstration that PRCC and KIN17 have roles in maintaining splice site identity during spliceosome assembly.

## Results

### The *C*. *elegans* allele *unc-73(e936)* can be used as a reporter of 5’ splice site choice

The *unc-73(e936)* allele has a G→U mutation at the 1st nucleotide (+1) position of the 16th intron. This mutation presents the spliceosome with an ambiguous 5’ splice site, resulting in the usage of two out-of-frame cryptic 5’ss and a striking uncoordinated phenotype [19] (Fig 1A). The majority of splicing (75%) occurs at a /GU dinucleotide found 23 nucleotides into the intron (the +23 site), resulting in an out-of-frame message. An additional 12% of splicing occurs at a position 1nt upstream of the wild-type splice site (the -1 site) using the new /GU dinucleotide formed by the *e936* mutation, also resulting in an out-of-frame message. We have previously demonstrated that these out-of-frame messages are not substrates for nonsense-mediated decay [19]. An additional 13% of splicing occurs at the wild-type splice site (the wt site), even though this defines an intron that begins with a non-canonical /UU. Only the small fraction of splicing at the in-frame /UU splice site produces full-length functional protein. The animals bearing the *unc-73(e936)* allele are able to live and reproduce through self-fertilization but are profoundly uncoordinated. Even a modest increase in splicing at the in-frame /UU splice site results in a dramatic phenotypic reversal which is visible at the plate level, making this allele a sensitive assay of perturbations to splice site choice. Using this screen, our lab has identified new extragenic suppressors over several iterations [16,17,19]. Because those three previous iterations of the *unc-73(e936)* suppressor screen have identified mutations on residues modeled near the active site of the spliceosome, and those mutations often change global 5’ splice site choice, we concluded that a genetic screen using this allele can identify loci that are capable of affecting splice site choice. Because we have never found the same extragenic suppressor mutation twice in 500,000 mutagenized genomes screened previously, the screen is not yet saturated. Therefore, we performed the genetic screen again to search for more suppressor mutations in splicing factors capable of altering splice site choice.

In a recent iteration of the *e936* extragenic suppressor screen, we recovered four new extragenic suppressor alleles with improved locomotion and a novel change in cryptic splicing. Using Cy-3 labeled primers in reverse transcription-polymerase chain reaction (RT-PCR) visualized after denaturing gel electrophoresis, we found that these four strains displayed a different pattern of cryptic 5’ splice site usage in *unc-73(e936)* compared to wild type, but, curiously, also a different pattern compared to previously identified modifiers [16,17,19]. While previous suppressors have reduced splicing at the +23 splice site with coordinated gains at both the -1 and wt sites, these four new suppressors had the most dramatic effect in altering the relative usage of the -1 and wt sites relative to each other, resulting in increased wt splice site usage to ~25% of *unc-73* messages, consistent with the improved locomotion phenotype identified in the screen (Fig 1B). We now refer to extragenic suppressors in three classes: Type I is the U1 snRNA suppressor *sup-39*, while Type II includes the protein factor suppressor alleles *snrp-27* (M141T) and *prp-8* T524S and G654E. The Type I and Type II suppressors both reduce +23 splice donor usage with concomitant increases in both the -1 and wt splice sites. The dramatic change in the relative usage of the -1 and wt sites is the key feature of these new Type III suppressors. In total, from all iterations of this screen performed in our lab we have screened 750,000 mutagenized genomes and recovered all motile worms and identified 10 extragenic and 11 intragenic suppressors. The Type I suppressor, some Type II suppressors and one intragenic suppressor have been characterized in published work [20,16,17].

### The four new Type III suppressor alleles are in the *C*. *elegans* homologs of KIN17 and PRCC

Using Hawaiian strain SNP mapping [23], as described in Methods, we mapped each of these four new suppressor alleles to an arm of a chromosome. Then, using high throughput DNA sequencing of the strain genomes, followed by SNP identification protocols to identify differences in genomic sequence from the starting *unc-73(e936)* uncoordinated strain (see Methods), we identified spliceosome-associated proteins and RNA binding proteins with mutations in their sequence within the chromosomal region.

**Fig 2:**
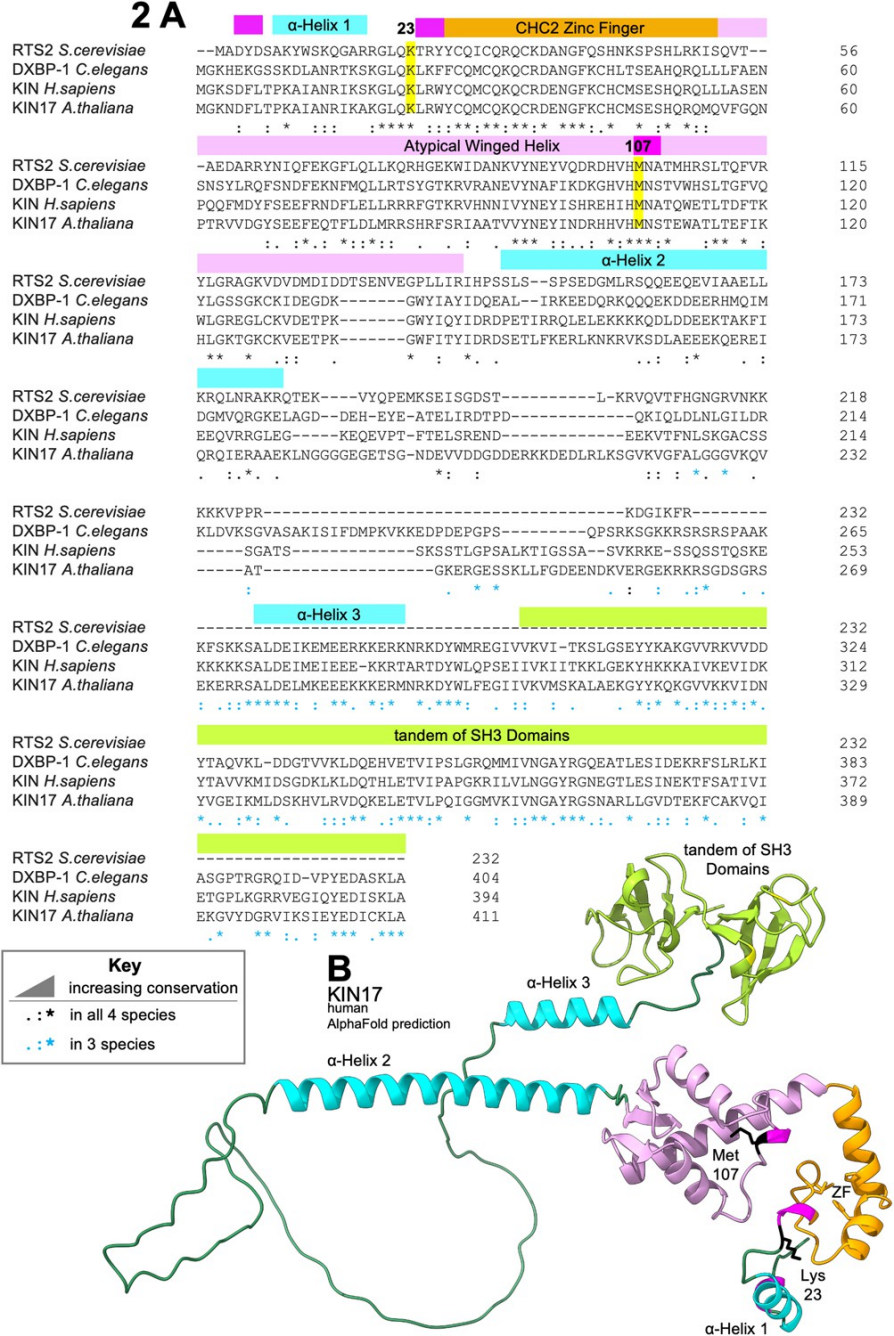
N-terminus of KIN17 is Highly Conserved Between Yeast, Worm, Human and Arabidopsis. A. Multiple sequence alignment of KIN17 and orthologs. 3^10^ turns in magenta, numbered α-helixes in cyan, residues K23 and M107 are highlighted in yellow, the zinc finger in orange, the atypical winged helix in orchid pink, and the tandem of SH3 domains in light green. Sequence conservation is annotated as described in the key. Alignment generated in Clustal Omega [25]. B. AlphaFold predicted structure of human KIN protein [24], colored to match the multiple sequence alignment above. Lysine 23 and Methionine 107 in black.

Two of the suppressor alleles had point mutations in the gene *dxbp-1*, the worm homolog of KIN17: a mutation that changes the 23rd amino acid from a lysine to an arginine (K23N, *az105*, Fig 1B, Lane 3) and another that changes the 107th amino acid from a methionine to an isoleucine (M107I) (*az33*, Fig 1B, Lane 4). Both of these residues are conserved between worm, human, yeast, and Arabidopsis (Fig 2A). *C*. *elegans dxbp-1*, or *dox-1*, is the homolog of a human and mouse gene known as KIN or KIN17. It is *not* a kinase. Except in the multiple sequence alignment (Fig 2A), throughout this manuscript, we will refer to KIN17 when talking about the protein, and *dxbp-1* when talking about the gene. K23 is adjacent to a CHC2 domain; the structure of the CHC2 domain of KIN17 has never been experimentally determined but is modeled in the AlphaFold [24] predicted structure (Fig 2B, orange). The 107th residue of the worm homolog of KIN17 resides in a 3_10_ helix on a loop in the atypical winged-helix domain (Fig 2B, orchid pink) [25]. This domain is “atypical” because the cluster of residues that are typically positively charged and coordinate nucleic acid binding in a winged-helix is not charged, leading to the hypothesis that the highly conserved 3_10_ helix is instead involved in protein binding [25]. KIN17 is predicted to have a disordered central region flanked by α-helices [15] (Fig 2B, cyan), followed by a tandem of SH3-like domains separated by a flexible linker (Fig 2B, light green) [26].

KIN17 was first identified in a search for mammalian homologs of the bacterial DNA repair protein RecA and has since been studied primarily for roles in DNA damage repair and transcription in eukaryotic cells [26–36] or cancer [37,38]. In *S*. *cerevisiae*, there is a named gene, RTS2, that shares homology with the N-terminal portion of KIN17 [39]. Observations about KIN17 include the following: KIN17 binds to single-stranded and double-stranded DNA [36,40–44] with a preference for AT-rich curved double-stranded DNA [30,45,46] and binds to RNA, with domains exhibiting preferences for specific poly-nucleic acid oligos [47,48]. KIN17 also binds to proteins in complexes of high molecular weight, including ones involving chromatin [40,44,49], DNA recombination [45], DNA damage repair [50], DNA replication [35,43], pre-mRNA splicing [47,51–54, 15], and translation [44]. It is likely that KIN17 performs more than one role in the eukaryotic cell.

**Fig 3:**
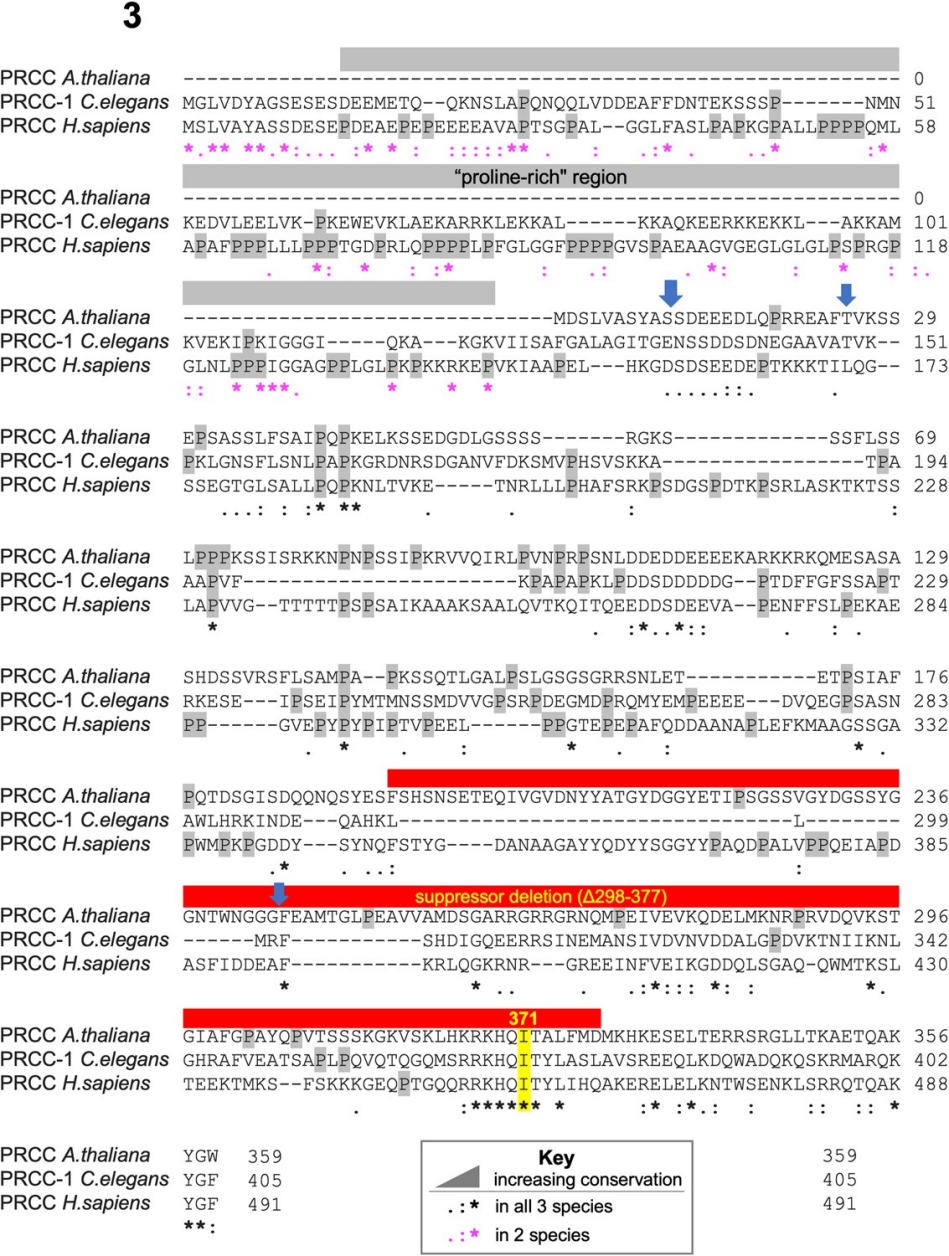
Both suppressor mutations overlap with the longest stretch of identity in PRCC. Multiple sequence alignment of PRCC-1 and orthologs. The “proline-rich” region frequently observed in human oncogenic fusions is indicated in gray, and all prolines are highlighted in gray, the suppressor mutation I371 is highlighted in yellow, the suppressor deletion (Δ289–377) is indicated in red. Sequence conservation is annotated as described in the key. PRCC(null) is not represented because it is a deletion of all coding regions of the gene. Vertical blue arrows mark the commonest breakpoints for PRCC N-terminal oncogenic fusions [74]. Alignment generated in Clustal Omega [97].

This screen also identified two mutations in *prcc-1*, the worm homolog of human PRCC: a mutation which changes the 371st amino acid from an isoleucine to a phenylalanine (I371F) (*az102* Fig 3), and a large deletion near the C terminus that removes amino acids 298–377 in frame (*az103*, Fig 3). Except in the multiple sequence alignment, throughout this manuscript we will refer to PRCC when talking about the protein and *prcc-1* when talking about the *C*. *elegans* gene. PRCC, known variously as proline-rich protein, proline-rich coiled coil, papillary renal cell carcinoma translocation-associated gene protein, and mitotic checkpoint factor protein, has been implicated in oncogenic fusions where the proline-rich N terminal region is fused to any of several transcription factors [55–57]. The proline-rich region is relatively proline-poor in *C*. *elegans* compared to human; the domain is absent in Arabidopsis. PRCC is predicted to be largely intrinsically disordered by AlphaFold, except for a few helixes near the C terminus [24]. The 371st amino acid of the worm homolog of PRCC occurs in the longest helix, in the middle of the longest stretch of identity, where 9 residues are conserved from worm to human. The deletion suppressor identified in this screen overlays that region, labeled by a red bar. (Fig 3). PRCC has been identified as a potential spliceosomal B^act^ complex component by mass spectrometry [58] and Yeast 2-Hybrid experiments [59].

To confirm that the three single amino acid substitution alleles identified by mapping and sequencing of the suppressor strains from the screen are indeed responsible for the altered cryptic splicing of *unc-73(e936)*, we used CRISPR/Cas9 to generate the same amino acid substitutions in wildtype worms (see methods) and tested these programmed alleles for an effect on the ratio of -1:wt splice site usage. The CRISPR-generated *prcc-1(az102)* allele can suppress *unc-73(e936)* splicing and movement defects, and alter cryptic splicing, confirming the identity of the PRCC(I371F) suppressor (Fig 1C, Lane 5). A deletion null allele of *prcc-1* generated by the *C*. *elegans* gene knockout consortium, *gk5556*, is viable and can both suppress the movement defects of *unc-73(e936)* and alter cryptic splice site usage (Fig 1C, Lane 6). This demonstrates that *prcc-1* is a non-essential gene and that loss-of-function leads to changes in splicing. The suppressor lines pulled out of the screen and all engineered suppressor lines tested in splicing are homozygous for their respective mutations in *prcc-1*.

Confirmation of the *dxbp-1* alleles by CRISPR is more challenging, as they map to the same chromosome as *unc-73*, making crosses difficult. On top of this, injection of CRISPR-cas9 RNP complexes into *e936* animals is challenging as the worms are sick and have smaller brood size. We solved this challenge by generating the two *dxbp-1* mutation alleles by CRISPR in a wild-type strain, followed by subsequent CRISPR mutation of *unc-73* to mimic the *e936* allele. These strains resulted in suppression of *unc-73* uncoordination and the predicted change in -1:wt splice site usage (Fig 1C, Lanes 4 and 5). In various genetic crosses, we were able to identify F1 animals heterozygous for the suppressor mutations and homozygous for the *unc-73(e936)* allele by their improved locomotion relative to unsuppressed *unc-73* mutant worms. These presumed heterozygous animals with improved movement were able in the next generation to produce offspring homozygous for suppressor mutation. This indicated to us that the point mutation Type 3 suppressor alleles are semi-dominant. To understand whether KIN17 is an essential gene, we used our standard CRISPR pipeline to generate a *dxbp-1(null))* allele (see methods). We put the *dxbp-1(null)* allele over a fluorescent hT2 balancer, designed such that homozygous *dxbp-1(+)* animals are GFP+ but homozygous lethal, heterozygous animals are GFP+, and animals homozygous *for dxpb-1(null))* do not fluoresce. We found that KIN17 deletion is embryonic lethal in *C*. *elegans*; occasionally GFP- animals homozygous for *dxbp-1(null))* can survive to something resembling L3 stage, however, these rare animals are severely underdeveloped and do not live to molt again. Simultaneously, the *C*. *elegans* Deletion Mutant Consortium [60] created a *dxpb-1(null))* allele and also found the deletion of *dxbp-1* to be homozygous lethal. This demonstrates that *dxbp-1* is an essential gene in *C*. *elegans*.

### KIN17 and PRCC promote usage of a non-canonical /UU 5’ splice site in 2-choice and 2x2-choice reporters

**Fig 4:**
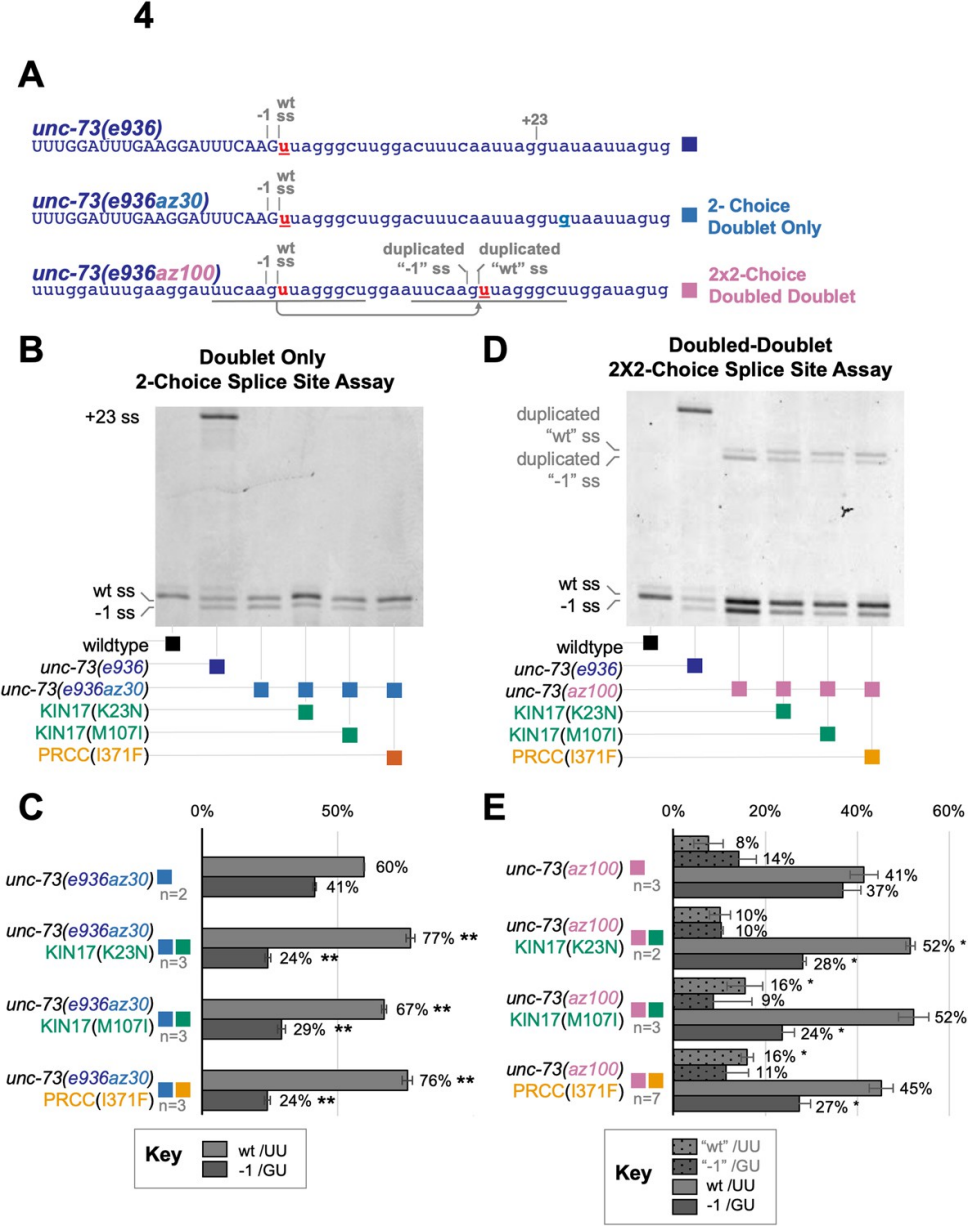
UU/ preference is independent of splice site location. **(A)** Sequences of three splice site choice competition reporters based on *C*. *elegans unc-73*: the first is the *unc-73(e936)* allele that allows for three cryptic splice sites as described in Fig 1A; below that, *unc-73(****e936az30****)* intragenic suppressor allele in which the +23 splice site is abolished by a A→G at the +26 position of the intron, leaving only the doublet of /G/UU splices sites, which we refer to as the 2-choice doublet-only splicing assay, and *unc-73(****az100****)* in which the genomic region of the doublet splice site has been duplicated, overwriting the downstream wild-type sequence and creating two /G/UU doublets, 18 bases away from each other, which we refer to as the 2x2 doubled-doublet splicing assay. **(B)** All three suppressors change the ratio of splice site usage at the doublet, promoting the /UU splice site. Poly-acrylamide gel showing Cy-3 labeled *unc-73* PCR products from cDNA. The alleles found in each sample are indicated in the figure. The same PCR primers are used on all samples; band positions and intensities are indicative of relative use of the available 5’ splice sites. **(C)** Quantification of PSI of the indicated strains. Error bars show Standard Deviation One. Star indicates p-value less than 0.05, and two stars indicate p-value less than 0.005 by Student’s 2-tailed T-test for samples with unequal variances when PSI for that splice site is compared to PSI in unc73(e936az30) control. **(D)** All three suppressors change the ratios of splice site usage at both the original doublet and the duplicated doublet, promoting the /UU splice site. Poly-acrylamide gel showing *unc-73* Cy-3-labeled PCR products from cDNA from the indicated strains with the indicated alleles. **(E)** Quantification of PSI of the indicated strains; details in Methods. Error bars show Standard Deviation. Star indicates p-value less than 0.05, by Student’s 2-tailed T-test for samples with unequal variances, when PSI for that splice site is compared to PSI in *unc73(az100)* control.

We were interested in the unique suppressive phenotype displayed by the mutations in KIN17 and PRCC, as they are so similar to each other but distinct from previously identified suppressor phenotypes in that they change the relative 5’ss usage of overlapping /G/UU splice sites. To investigate this further, we utilized an intragenic suppressor allele of *unc-73*, *e936az30*, in which an A→G mutation at the +26 position of the intron eliminates the usage of the +23 cryptic splice site (Fig 4A). Therefore, the only two splice sites available are the cryptic /GU and the non-canonical /UU one nucleotide downstream; we refer to it as a 2-choice splice substrate. In a wild-type background, these two splice sites are used about 41% and 59% of the time, respectively (Fig 4B, Lane 3). In a KIN17(K23N), KIN17(M107I), or PRCC(I371F) background, we see altered ratios of splice site use in the 2-Choice splice site competition assay relative to wild-type background (Fig 4B). The splicing pattern was similar in the presence or absence of the + 23 /GU splice site (compare with Fig 1C). Despite the /GU being the primary hallmark of the 5’ splicing landmark, these suppressor alleles are promoting usage of the adjacent /UU 5’ss. In the KIN17(K23N), KIN17(M107I), and PRCC(I371F) strains, the relative /UU splice site usage is increased to 77%, 67%, and 76%, respectively (Fig 4B and 4C). When the percent spliced in (PSI) for the UU splice site in mutant strains was compared to the control strain, all three suppressors were found to have highly significant p-values by student’s t-test. Those test statistics are reported in S2 Table.

In the 2-Choice splice site competition assay, we found that mutations in PRCC and KIN17 promote usage of a non-canonical /UU splice donor over an adjacent upstream /GU splice site. We wondered whether the information to promote /UU splicing was contained within the 5’ss itself, whether it was promoted by some nearby splicing enhancer element, or whether it was dependent on a distance from the original splice site. To answer these questions, we devised a new competition assay that would separate sequence from location. Using CRISPR/Cas9 and a repair oligo, the region bearing the curious /G/UU 5’ss doublet was duplicated in the native *unc-73* gene, and inserted downstream, overwriting the downstream bases of the intron (Fig 4A, allele *az100*). This doubled the splice donor doublet, creating a 2x2-choice splice site assay, featuring two 2-choice splice site doublets separated by 18 bases. We knew the second doublet was close enough to be chosen by the spliceosome because it was proximal to the + 23 site from the 3-choice splice site assay in the original *unc-73(e936)* allele. We abolished the + 23 splice site so that only the four choices contained in the two doublets remained. In a wild-type background, both splice sites of the original doublet are used more than either of the splice sites in the duplicated doublet downstream. In the upstream doublet, there is a slight preference for the /UU splice site (53%), while in the less-used downstream doublet the /UU site is less-preferred (34%) (Fig 4D, Lane 3).

When this “doubled-doublet” *unc-73(az100)* allele is combined with suppressor alleles KIN17(K23N), KIN17(M107I), or PRCC(I371F), we see altered ratios of splice site use in the 2x2-Choice splice site competition assay relative to wild type (Fig 4D). In all three cases, both doublets are used, and, similar to control, most splicing comes from the upstream doublet. In the presence of any of these three suppressor alleles, the usage of the /UU splice site increases relative to the /GU splice site in both the original doublet and the duplicated doublet, 18 nucleotides downstream. The percentage of splicing at the original -1 /GUU site is significantly reduced in mutant versus control (Fig 4E); p-value assessed by Student’s t-test. Those test statistics are reported in S3 Table. When the ratio of splice site usage at each doublet is considered independently, for KIN17(M107I) and PRCC(I371F) we see that at both doublets, usage of the /UU splice site is significantly increased (Fig 4E). In KIN17(K23N) the increase in usage of the original/UU site, but not the duplicated site is statistically significant (S3 Table). These data support the hypothesis that the information for switching to /UU splice donor usage in the presence of these suppressor alleles is dependent on the 5’ss sequence and not a distance from some other markers on the pre-mRNA.

### Analysis of splicing changes in native genes in the presence of KIN17 and PRCC suppressor alleles

Because mutations in KIN17 and PRCC can promote usage of 5’ /UU splice sites in our splice site competition assays, we wanted to know if those mutations also changed splice site choice at native loci. The *unc-73* transcript, upon which all of our splice site competition assays are built, is not subject to nonsense-mediated decay [19], which is why we can recover cryptically-spliced frame-shifted transcripts. However, when looking for alterations displaying site choice more broadly, we expect that most transcripts will be targeted by nonsense-mediated decay (NMD), especially given that the prominent splicing change we might expect to see would move the start site of an intron over by a single nucleotide, thus changing the reading frame. Given that, it might be difficult to detect these changes in splicing as they may potentially lead to differential transcript stability. *C*. *elegans* is a rare metazoan able to survive without a functional NMD pathway, making it possible to experiment in an NMD knockout background [61]. We designed a CRISPR/Cas9 engineered *smg-4* null allele, *az152*, which is easily detectable by single worm PCR and restriction digest, allowing for ease of mapping in crosses; *smg-4* was chosen for creating an NMD mutant strain as it is not located on the same chromosome as *dxbp-1* or *prcc-1*. We confirmed that the new *smg-4* allele is NMD-defective by both the presence of the protruding vulva phenotype and the accumulation of NMD-targeted isoforms of *rpl-12* (S1 Fig) [62].

We used genetic crosses to create strains with KIN17(K23N), KIN17(M107I), PRCC(I371F), or PRCC(null) combined with *smg-4(az152)*, isolated mixed-stage mRNA, and performed mRNA-seq on three biological replicates for each suppressor strain, as well as on the original *smg-4(az152)* mutant strain as a control; 15 libraries in total. We performed 75x75nt paired-end reads and obtained between 46M and 69M reads for each library. We performed STAR mapping, which we modified to accommodate /UU 5’ splice sites as described in Methods. Briefly, this modification to STAR protects against the program’s bias towards canonical splice sites, which might otherwise cause us to miss true alternative splice sites with non-canonical intron starts such as UU. We ran an alternative splicing analysis which looked at both annotated and unannotated alternative 5’ and 3’ splicing events, as well as Ensembl-annotated skipped exon, mutually exclusive exon, multiply skipped exon, intron inclusion, alternative first exon, and alternative last exon events. For each alternative splicing event, we quantified relative usage of each junction in each of the 15 libraries (percent spliced in or PSI). We then compared the ΔPSI for each event between each library and the starting *smg-4* mutant strain. We performed pairwise comparisons between each of the three biological replicates of a suppressor strain against each of the three biological replicants of the control NMD mutant strain alone, for a total of 9 pairwise comparisons for each alternative splicing event, and asked how many of those 9 comparisons generated a ΔPSI of >15%. Those events for which all 9 pairwise comparisons had a ΔPSI >15% (pairSum = 9) were then analyzed individually on the UCSC Genome Browser with the RNASeq tracks [63] to confirm the alternative splicing event. We then filtered these confirmed pairSum = 9 events for those where there was a >20% average ΔPSI across the 9 pairwise comparisons. Table 1 summarizes the number of confirmed alternative splicing events meeting these strict criteria in each strain comparison. Detailed annotations and locations for the alternative 5’ and 3’ splicing events are shown in S4 Table.

**Table 1:** Type III Suppressors have Variable Effects on Genome-Wide Alternative Splicing.

pairSum = 9 with Minimum ΔPSI = 0.15 & Average ΔPSI >0.20 (n = 9)	KIN17 (K23N)	KIN17 (M107I)	PRCC (I371F)	PRCC (null)
	SZ340 vs. SZ345	SZ340 vs. SZ355	SZ340 vs. SZ346	SZ340 vs. SZ356
Alternative 5’ Events	**4**	**3**	**69**	**90**
Alternative 3’ Events	**108**	**24**	**1**	**35**
Skipped Exons	**7**	**2**	**0**	**5**
Retained Introns	**2**	**0**	**2**	**1**
Multi Skipped Exons	**0**	**0**	**0**	**0**
Mutually Exclusive Exons	**1**	**0**	**0**	**0**
Alternative First Exons	**5**	**1**	**0**	**5**
Alternative Last Exons	**7**	**1**	**0**	**1**

### PRCC(I371F) and PRCC(null) promote usage of 5’ /UU splice sites and adjacent 5’ /GU splice sites throughout the *C*. *elegans* transcriptome

**Fig 5:**
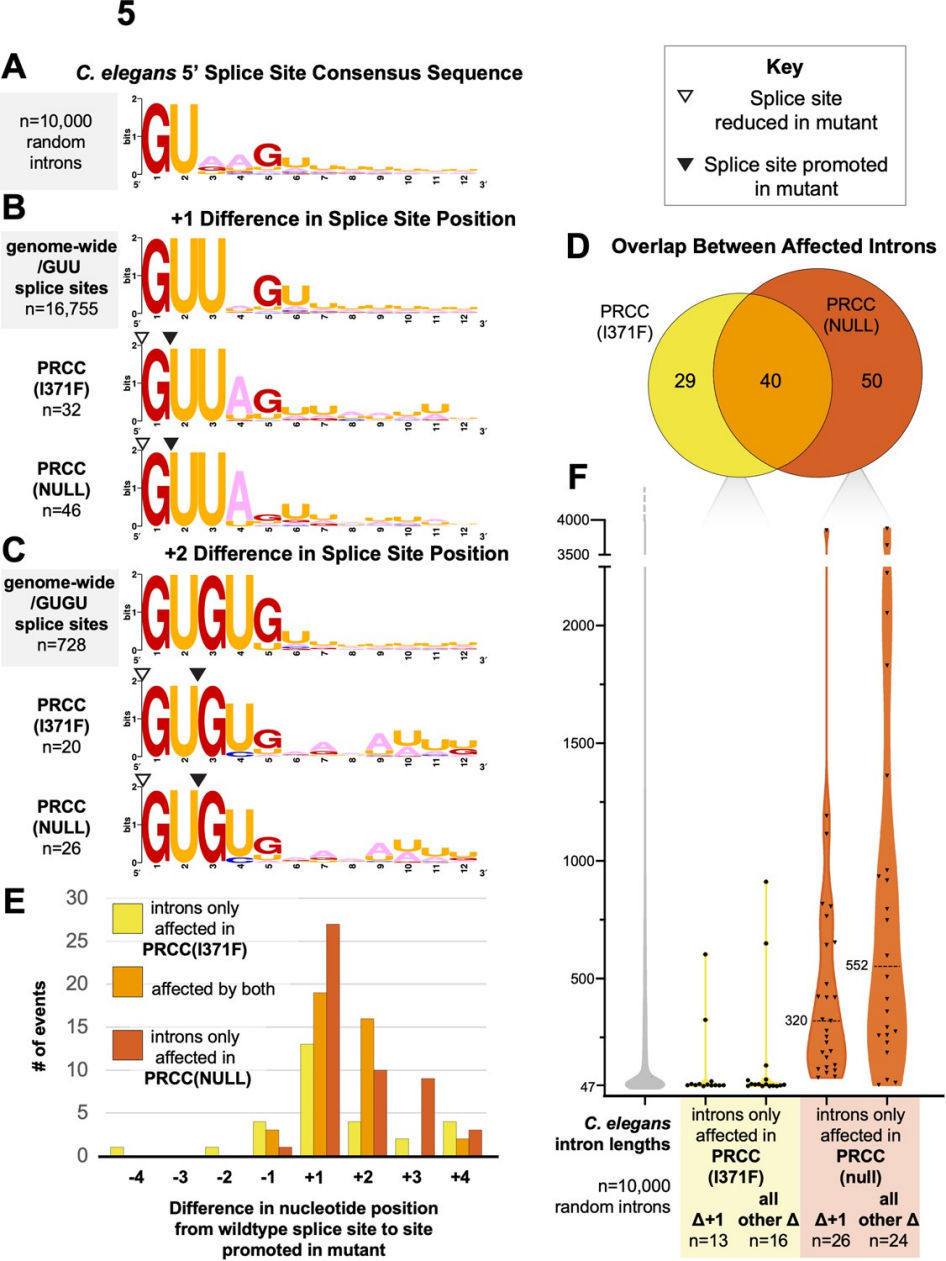
Throughout the genome, mutations in PRCC increase usage of /UU 5’ splice sites and /GU 5’ splice sites lacking other features. **(A)** Sequence logo showing the consensus sequence for the 5’ end of 10,000 randomly chosen *C*. *elegans* introns. **(B)** Sequence logo of introns that are differentially spliced in PRCC mutant backgrounds and follow the /G/UU splicing pattern seen in *unc-73(e936)* compared to all annotated introns that begin with /GUU. The splice site promoted in mutant is +1 nucleotides from the position of the predominant /GU splice site. Splice sites are indicated by triangles, as described in the key. **(C)** Sequence logo of introns that are differentially spliced in PRCC mutant backgrounds in which the splice site promoted in mutant is +2 nucleotides from the position of the predominant /GU splice site. Splice sites are indicated by triangles, as described in the key. **(D)** Euler diagram enumerating the overlap between affected introns differentially spliced in the presence of the two PRCC alleles. **(E)** Most splice sites promoted by the PRCC alleles are either one or two nucleotides downstream of the predominant splice site. Frequency and direction of nucleotide shift between the splice site favored in wild type, and the splice site promoted in PRCC mutant. **(F)** Violin plot showing the lengths of introns affected only in a given suppressors group. The five violins correspond to: 10,000 random wild-type *C*. *elegans* RefSeq introns, the subset of 13 introns in PRCC(I371F) in which the splice site promoted was at a /UU splice site 1 nucleotide downstream from the predominant splice site, the 16 affected introns in that same strain that did not follow +1 pattern, the subset of 26 introns in PRCC(null) in which the splice site promoted was at a /UU splice site 1 nucleotide downstream from the predominant splice site, the 24 affected introns in that same strain that did not follow +1 pattern. These groups of introns have median lengths of 47, 48, 51, 320, and 552 nucleotides, respectively.

Using the stringent criteria described above, we were able to identify multiple examples of changes to 5’ splicing in the presence of PRCC mutations. In PRCC(I371F) and PRCC(null), we found, respectively, 34 and 46 examples of introns where mutant strains promote usage of a downstream /UU splice site over an adjacent /GU splice site (Fig 5B). This type of intron start of /G/UU 5’ splice site is similar to the *unc-73(e936)* splice site choice competition assays. Similarly to the *unc-73* intron, which has an A in the 4th position, these affected introns are enriched for an A in the 4th position of the intron immediately following the GUU (Fig 5B). Unlike the *unc-73* intron, which has a G in the 5th position, the introns affected by PRCC(null) show less dependence on a G in the 5th position (Fig 5B). Fifty-eight percent of the introns affected by PRCC(I371F) are also affected by PRCC(null) (Fig 5E).

In PRCC(I371F) and PRCC(null), background, we also found 37 and 44 instances, respectively, of events where the alternative 5’ splice site promoted in the presence of PRCC mutations were at /GU dinucleotides, either 2,3, or 4 nucleotides away from the wild-type /GU dinucleotide. Most of these shifted downstream (Fig 5E). A substantial portion of the introns affected by the PRCC-1(null) were also affected by the point mutation in PRCC(I371F) (Fig 5D). Surprisingly, despite the similarity between the splicing phenotypes observed in our *unc-73(e936)*-based splice site competition assays for both PRCC and KIN17 mutations, we found few examples of changes to 5’ splice site choice at endogenous introns in the presence of either of the two KIN17 mutant alleles using the stringent criteria employed for Table 1.

### PRCC null affects alternative 5’ splicing at longer introns

We were interested in the group of introns affected by PRCC mutations, so we looked at the lengths of introns, and flanking exons. Despite the overlap between affected introns, the average intron length for each group is very different. Because rare, very long introns can exert a strong influence on averages, we report the median intron length. To focus more on the relative contribution to median intron length in each category, we removed events in common and looked at the lengths of introns unique to each dataset (Fig 5D). While the median intron length for /UU and /GU alternative splice sites promoted in PRCC(I371F) background is similar to the overall median intron length in *C*. *elegans* of 51 nucleotides [64], the median intron length of PRCC(null) promoted alternative introns for both /UU and /GU introns is much longer, with a median length of 320 and 552 nucleotides respectively (Fig 5F).

### KIN17(K23N) and KIN17(M107I) affect 5’ splice site in a similar manner to PRCC mutations, but with a smaller effect size

**Fig 6:**
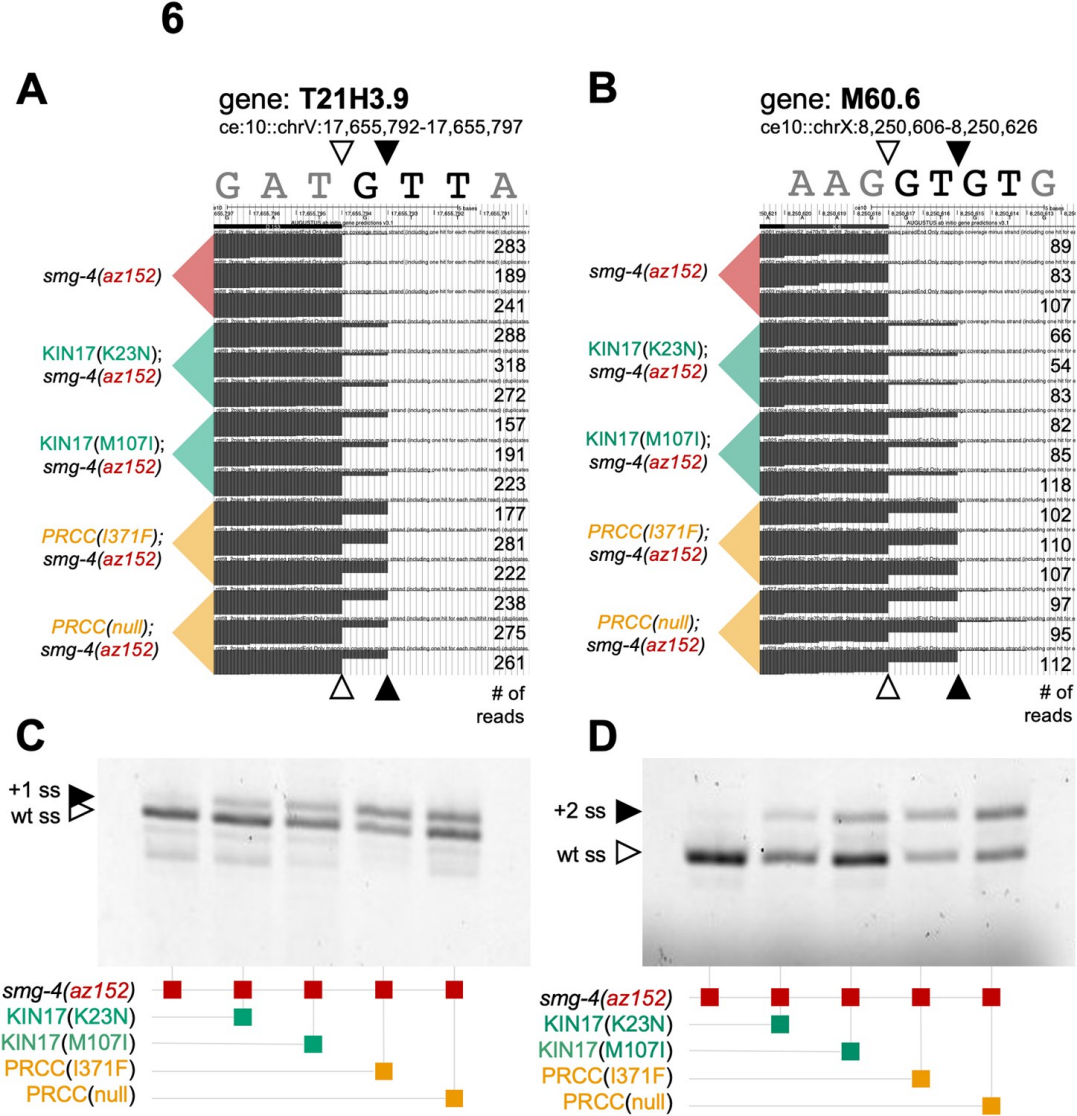
KIN17(K23N) and KIN17(M107I) affect 5’ splice site in a similar manner to PRCC mutations, but with a smaller effect size. **(A)** UCSC Genome Browser shot of RNA-seq coverage tracks at the gene T21H3.9. For each of five strains indicated on the left-hand side, three replicates are visible for each, the number of reads supporting the track is on the right-hand side. White triangles indicate the wildtype splice site reduced in mutant; black triangles indicate the alternative splice site promoted in mutant. The 5’ splice site switching in KIN17 is above wildtype levels but did not meet our strict criteria for inclusion in Table 1. **(B)** UCSC Genome Browser shot of RNA-seq coverage tracks at the gene M60.6. For each of five strains indicated on the left-hand side, three replicates are visible for each, the number of reads supporting the track is on the right-hand side. White triangles indicate the wildtype splice site reduced in mutant; black triangles indicate the alternative splice site promoted in mutant. The 5’ splice site switching in KIN17 is above wildtype levels but did not meet our strict criteria for inclusion in Table 1. **(C)** Verification of RNA-seq results showing that KIN17 mutations switch 5’ ss, just not as strongly as PRCC mutations. Image is a scan of a denaturing poly-acrylamide gel showing Cy-3 labeled T21H3.9 PCR products from mixed-stage cDNA. **(D)** Verification of RNA-seq results showing that KIN17 mutations switch 5’ ss, just not as strongly as PRCC mutations. Image is a scan of a denaturing poly-acrylamide gel showing Cy-3 labeled M60.6 PCR products from mixed-stage cDNA. Quantification of three biological replicates of the gels in parts C and D are provided in S5 Table.

We chose to confirm two of alternative 5’ss events identified for by mRNASeq by reverse transcription-PCR. We chose one example each of a G/UU alternative event and a GU/GU alternative event, based on the coverage tracks for the 15 mRNA-Seq libraries for these two regions shown in Fig 6A and 6B. Note that while the switch to the downstream 5 ‘splice site is strong in the PRCC mutants as expected from the mRNA-seq data, we also see evidence that the KIN17 mutants have increased usage of the downstream 5’ss relative to the control strain, despite the fact that these splicing events were not called by our analysis pipeline for either KIN17 mutant. Fig 6C and 6D show representative RT-PCR products for these two alternative 5’ splicing events for the 5 strains, and these confirm the results from the mRNA-Seq data (quantitation for three biological replicates of the experiments in Fig 6C and 6D are found in S5 Table). Not only do the PRCC mutant strains show the predicted splicing change, but the KIN17 mutant strains also show a detectable, but weaker, switch to usage of the downstream alternative 5’ss. For the G/UU event in T21H3.9, the KIN17(K23N) mRNASeq analysis only showed a pairSum = 3 for a 15% ΔPSI in the 9 pairwise comparisons to the control, while for KIN17(M107I) there was pairSum = 9, but the mean of the 9 ΔPSI was 19.7%, just below the 20% cutoff used for Table 1. For the GU/GU alternative 5’ splicing event in M60.6, the KIN17(K23N) libraries had a pairSum = 0, indicating that all comparisons were below 15% ΔPSI, while for KIN17(M107I) mRNASeq libraries, we measured pairSum = 8, indicating that one of the pairwise comparisons to the control strain had a ΔPSI less than 15%. These RT-PCR results, combined with the mRNASeq studies on these two events, indicate that the KIN17 mutants may have more alternative 5’ss targets than are reported in Table 1.The PRCC mutants have strong effects on many native targets while the KIN17 mutants may have weaker but detectable effects on these same splice sites. Most of the alternative 5’ss events called by RNA-seq analysis in KIN17(K23N) and KIN17(M107I) mutants are also found in both PRCC mutants. Two of these target introns, in *spas-1* (a /G/UU type) and *cec-10* (a /GU/GU type), are found in all four suppressor mutants using the stringent criteria employed for listing in Table 1. These results indicate that both KIN17 mutants may cause a similar change in 5’ splice site sequence preference as the PRCC mutants. However, the KIN17 mutants cause a smaller ΔPSI.

### KIN17 3’ splicing changes appear to be an indirect effect caused by changes to population dynamics

Surprisingly, KIN17 mutations, identified in a screen for modifiers of 5’ splice choice, with only modest effects on genome-wide 5’ss choice, our mRNASeq pipelines called many instances of 3’ splice site choice. The 3’ splice sites promoted in the RNA samples with KIN17 mutations were highly degenerate sites (S2A Fig), mostly located in-frame, 6 or 9 base pairs away, and unidirectionally upstream of the adjacent consensus 3’ splice sites (S2B Fig). We found 108 examples of alternative 3’ss usage in KIN17(K23N), 24 examples in KIN17(M107I), and 35 examples in the PRCC(null) (Tables 1 and S4). Most of the intron events identified in KIN17(M107I) were also represented in the KIN17(K23N) events (S2C Fig). We found only 5 unique examples of PRCC(null) mutations affecting 3’ splice site choice that are not shared with the KIN17 mutant strains. The unidirectional shift to a poor consensus upstream 3’ss is highly similar to developmentally regulated alternative splicing events in which cells in the *C*. *elegans* germline show more splicing to an upstream, poor consensus alternative 3’ss relative to somatic cells [64]. In that study, 203 alternative 3’SS events were identified as being developmentally regulated; 49 of those alternative 3’ splicing events overlap with the alternative 3’ splicing events identified in PRCC and KIN17 mutants (S2D Fig).

**Fig 7:**
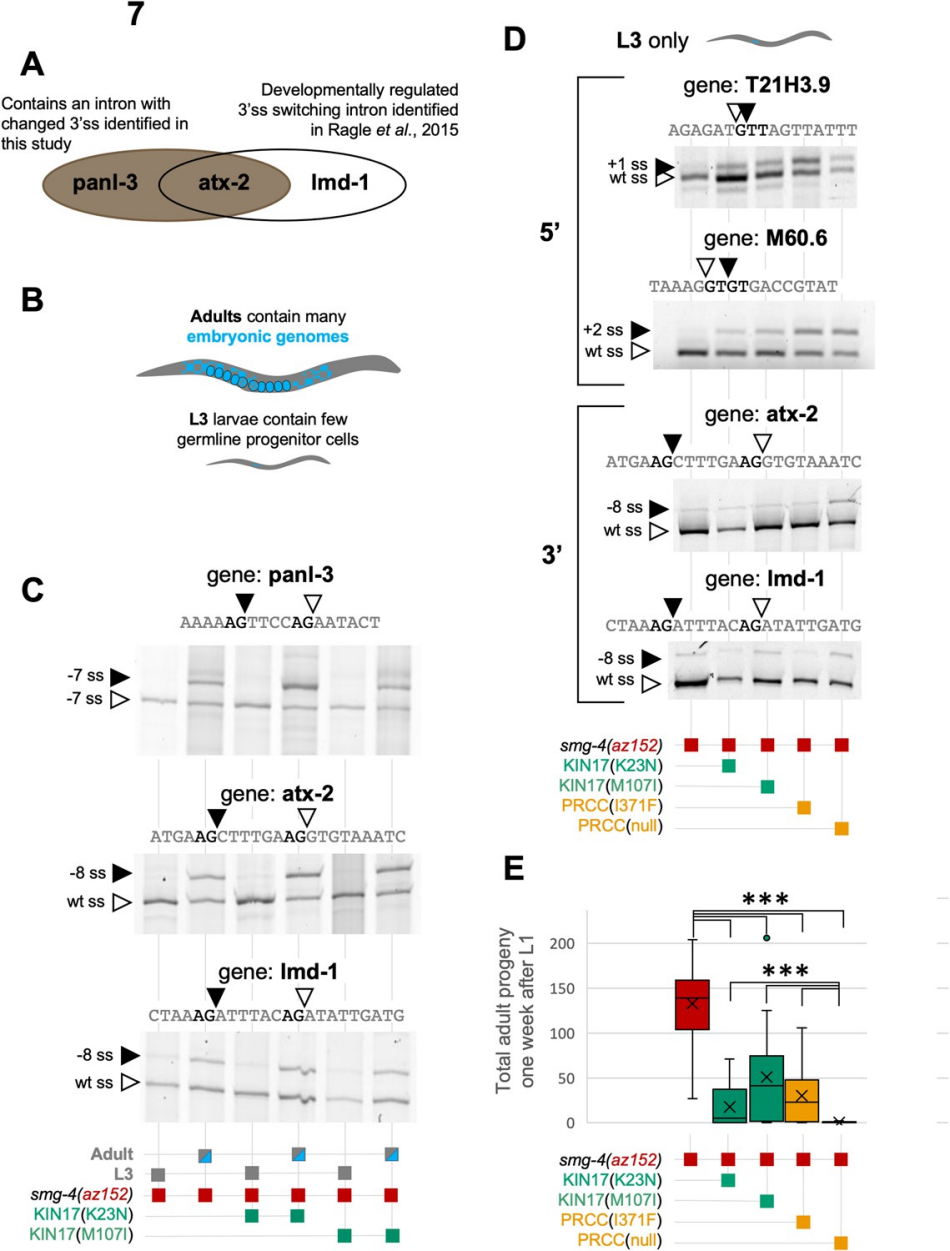
KIN17 and PRCC(null) mediated alternative 3’ splicing is caused by the ratio of embryos in a mixed stage population. **(A)** 3 example genes with which we chose to differentiate between embryonic-type splicing and somatic-type splicing. **(B)** An adult hermaphrodite *C*. *elegans* contains roughly as many embryonic genomes as somatic genomes (~1000 for each type), while L3 larvae have about the same number of somatic cells, but only about 50 embryonic precursor cells. **(C)** Reverse transcription and PCR results for three different alternative 3’ splicing events from staged L3 and adult animals from smg-4 control strain and strains containing smg-4 and either KIN17(K23N) or KIN17(M107I). The genomic location of the events is listed above each gel and the developmental stage and genotypes are listed below each gel. Quantification of three biological replicates of these gels is provided in S6 Table. **(D)** RT-PCR results from a control and four different suppressor strains (indicated below the figure) on synchronized L3 animals. The top two reactions are from alternative 5’ splicing events and the bottom two reactions are from alternative 3’ splicing events. Quantification of three biological replicates of these gels is provided in S6 Table. **(E)** KIN17 and PRCC mutants alter population dynamics. Box and whisker graph of total adult progeny of a single L1 animal after one week. By T-test, all four mutants show p<0.0001 compared to control, and the three point mutations show p<0.0001 when compared to PRCC(null) (S7 Table).

The overlap between the alternative 3’ splicing events identified in mRNASeq for the KIN17 and PRCC mutants with our previously reported germline-specific alternative 3’ splicing events [64], especially in regards to the unidirectionality of alternative splicing changes, led us to look more closely at whether these changes are the direct result of alternative splicing at the level of the spliceosome or result from changes in population dynamics that would change the relative amount of germline tissue in a mixed-stage culture. We tested three alternative 3’ splicing events, that were identified either in mRNASeq of mixed stage cultures in this experiment (*panl-3* and *atx-2*) and/or were known to be developmentally regulated in the germline (*atx-2* and *lmd-1*) (Fig 7A). We measured alternative splicing in RNA derived from synchronized L3 animals, which only contain ~48 germ nuclei in their small developing gonad, or synchronized young adult animals, which contain ~676 germ nuclei in their expanded gonads [65]. The germline size differences between adults and L3s are shown in Fig 7B in cartoon form. Strikingly, for all three alternative 3’ splicing events tested in the control strain or the two KIN17 mutant strains, we saw no difference among the strains in the usage of the alternative 3’ splice sites (Fig 7C). All were under developmental control with L3s preferring the distal 3’ss and adults switching to usage of both sites. This result was surprising because the mRNASeq results for alternative 3’ splicing events from KIN17 mutant strain K23N would suggest that we should see a change in splicing at all stages, yet in synchronized animals, the results are the same as the controls. This suggested that the alternative 3’ splicing changes that we saw in mRNASeq of mixed stage cultures were not directly caused by the KIN17 mutants but perhaps were the result of changes in population dynamics in the mutant strains, and the germline-specific alternative 3’ splicing switch to the upstream site that we observed in mixed-stage cultures is a readout of those changes. In addition, this analysis showed that the alternative splicing event in *panl-3* should be added to the list of developmentally regulated alternative splicing events from the Ragle *et al*. [64] study.

To further test this phenomenon, we isolated RNA from synchronized L3 animals from the same control, KIN17, and PRCC mutant strains that were used for mRNASeq. We tested several substrates for splicing changes between the strains. For the alternative 5’ splicing events for T21H3.9 and M60.6, the L3 RNA (Fig 7D) gave very similar results for changes in alternative splicing as the mixed stage RNA in Fig 6C and 6D (see S3 Table for quantitation over 3 biological replicates); the PRCC mutants had a stronger splicing change than the KIN17 mutants, but all had changes relative to the control strain. This indicates that the alternative 5’ splicing events are not dependent on developmental staging for the mutant strains. This is consistent with our initial isolation of the KIN17 mutants as suppressors of 5’ cryptic splicing where phenotypic uncoordination suppression was seen at all growth stages. In contrast, for the alternative 3’ splicing events for *atx-2* and *lmd-1*, the mutants and the controls showed no differences in synchronized L3 larva, unlike in the mixed stage mRNASeq data (S4 Table) where we saw the *atx-2* splicing shift towards the upstream 3’ss relative to the control strain. These data suggest that while the changes in alternative 5’ splice site usage in the KIN17 and PRCC mutants are an authentic direct effect on splice site choice, the changes in the alternative 3’ splice site usage in the KIN17(K23N) mutants may be indirect and result from changes in population dynamics that alter the abundance of germline in the culture and thus the amount of alternative 3’ss usage associated with the germline.

We did another test to ascertain whether the KIN17(K23N) strain that showed alternative 3’ss usage on native genes in our RNASeq analysis of mixed stage RNA was due to changes in germline gene expression in the library. We used a DESeq analysis [66] to identify genes whose expression changes between the strains in the mRNASeq data. We identified the genes with significant changes in gene expression (adjusted p-value <0.1) and then we looked at the Tissue Enrichment Analysis [67] terms for the genes with the highest expression changes relative to the control strain (S3 Fig). Strikingly, for the KIN17(K23N) strain relative to the control strain, the most common tissue enrichment terms for genes with major expression changes were for “Germ Line” and “Reproductive System”. Given that the KIN17(K23N) strain had the most alternative 3’ splicing events, and that it is the strain whose mixed stage mRNA is most enriched in germline genes, and that germline expression is associated with changes in alternative 3’ splice site usage, this DESeq tissue enrichment analysis provides more evidence that the changes in native alternative 3’ss usage that we see in our mRNA Seq analysis may be due to changes in developmental dynamics in mixed stage populations.

We had noticed in culturing these animals that, while all strains were viable, some strains seemed to take longer to grow than others. To test the hypothesis that there are changes in population dynamics in the mutant strains, we next set out to measure viability and growth of these animals. Fig 7E shows the results of one of these experiments in which a single L1 from each strain was put onto a 6cm NGM agar plate and grown at 20C for one week. L1s were chosen for the initial plating as this would allow us to monitor whether all hatched animals had the ability to grow to fertile adults. Adult progeny of that L1 were counted after one week. All mutant strains had fewer progeny than the control strain, with the PRCC(null) strain showing the fewest progeny. Checked for statistical significance by student’s t-test, all four strains bearing mutant alleles were highly significantly different from control, with p values of less than 0.0001, and strains bearing KIN17(K23N), KIN17(M107I), PRCC(I371F), were highly statistically significant when compared to PRCC(null) (S5 Table).

In the specific case of these alternative 3’ splicing events identified in Table 1, it appears that changes in population dynamics in mixed-stage cultures between the strains, especially for KIN17(K23N), increase the number of germline cells in a mixed-stage population, thus increasing the use of germline-specific alternative 3’ splicing [64]. The use of RNAs from synchronized cultures helps to resolve that the alternative 5’ splicing events are due to direct effects on splicing, while the alternative 3’ splicing events are likely the result of changes in germline ratios in the mixed stage cultures that lead to enrichment of alternative 3’ splicing events (Fig 7). This is a challenge for us in trying to identify broad changes in splicing in a small animal not readily prone to dissection. We use mixed-stage RNA to survey the broadest number of genes for alternative splicing, but we need to be cognizant when we do so that the mutants do not change the relative amount of germline cells in the population, as the development of that tissue leads specifically to a dramatic expansion of alternative 3’ splicing events [64].

## Discussion

This work represents the first direct demonstration that KIN17 and PRCC have a role in splice site choice. Prior to this manuscript, KIN17 was classified in the Spliceosome Database under “misc. proteins found irregularly with spliceosomes” (http://spliceosomedb.ucsc.edu/proteins/11606, accessed 3/22/2021), and had been primarily studied for roles in DNA damage repair and cancer, not splicing. We report here that mutations in the N-terminal unstructured region (K23N) and in the winged-helix (M107I) of KIN17 promote usage of an unusual /UU 5’ splice site downstream of an adjacent /GU splice site (Figs 1 and 6). This demonstration of KIN17 as a bona fide splicing factor may potentially point to a closer association between pre-mRNA splicing and DNA damage repair than is currently understood. PRP19 is a multifunctional ubiquitin ligase known to be a component of both spliceosomal and DNA damage repair complexes [68], and a recent study showed that U1snRNP and components of the DNA damage response compete for binding at human 5’ splice sites [69]. As both splicing and DNA damage repair require the recognition, cutting, and joining of nucleic acid chains, it may not be too surprising that they share some factors in common.

Prior to our studies, PRCC had a firmer association to the spliceosome, identified as a factor in B^act^ complexes through Yeast two-hybrid and mass spectrometry experiments [13,59], but no functional role had been identified nor had it been modeled into any metazoan spliceosomal structures (there is no *S*. *cerevisiae* homolog of this factor). Given the high degree of predicted disorder [24], it is unlikely that PRCC will ever model into X-ray crystallography or cryo-EM structures; genetic analyses such as the data presented here are essential to understanding the function of intrinsically disordered proteins such as PRCC. We report here that an I371F point mutation, located in the 9-residue-long region in the C-terminus of PRCC that is identical between worms and humans, changes 5’ splice site choice at native loci, and that is a non-essential gene and that the null allele also promotes extensive changes in alternative 5’ splicing (Table 1). It is possible that PRCC is serving a different function in *C*. *elegans* than it does in other organisms; the “proline rich-region” of PRCC most often found in oncogenic fusions is noticeably proline-poor in the *C*. *elegans* homolog relative to humans. The identification of a suppressor point mutation in a conserved region of the C-terminus points to a potential key region for splicing control.

There are mutations in key spliceosomal proteins such as SF3b1 and SR proteins, that are associated with cancer progression [70–72]. KIN17 upregulation has been shown to increase proliferation of lung and breast cancers [38,73] and knockdown of KIN17 reduces cell growth and increases cancer apoptosis [37]. Given the categorization of KIN17 as a DNA damage repair protein, these effects of KIN17 on cancer have been taken as evidence that KIN17 promotes genome stability. In patients with renal cell carcinoma, PRCC has been repeatedly found as part of oncogenic fusions, with the N-terminal proline-rich region of the PRCC gene fused to one of several transcription factor genes [55,56,74,75]. The oncogenic mechanism of these fusions is not known. Those oncogenic fusion breakpoints are indicated by blue arrows in Fig 3, with the anterior portion of the gene involved in the fusion product. That “proline-rich” region in humans contains 10 times as many prolines as in *C*. *elegans* and is predicted to be unstructured [24]. The PRCC point mutation we report here as driving changes in splice site choice in *C*. *elegans* is in the highly conserved C terminal region. The suppressor deletion found in our genetic screen overlaps with one oncogenic fusion region. Given the low conservation between the anterior region of PRCC between worms and humans, we find it unlikely that the mechanism of PRCC fusion oncogenesis is through association with the spliceosome.

The discovery of this new class of suppressors of *unc-73(e936)* cryptic splicing has led us to think about the splice site like a piece of evidence in a criminal case, held by “escorts” which shuttle the precise genetic landmarks through dramatic conformational changes. Each escort of the 5’ splice site, must by nature, hold it reversibly. Therefore, slipping or disengagement is possible while the 5’ss is in the custody of a snRNP or protein factor guardian, especially when the pre-mRNA is under tension from helicases or other components of the spliceosome. If we follow the chain of custody, we expect that translocations and changes of possession are likely to be inflection points where alterations to splice site identity, relative to the initial identification by early factors, are more likely. Some factors capable of affecting splice site choice may assist during those vulnerable moments in the splicing cycle. When an escort repositions or lets go entirely, these factors may make nucleotide shifts less likely. We see in the presence of the suppressor alleles identified in this study, that the spliceosomal components are choosing degenerate splice sites. The positions we have identified in KIN17 and PRCC may serve to prevent such slips in wild type during vulnerable points in the chain of custody. These mutations display a different splicing phenotype from previously identified suppressors. Instead of the predictable reduction of the distal +23 site and relatively even increase in usage of both splice sites of the doublet observed in factors previously identified (Fig 1D) [16,17], this new class of Type III suppressors displays a sharp change in the ratio of usage of the two adjacent splice sites of the doublet of adjacent splice sites, with the downstream /UU site promoted over the adjacent /GU site (Fig 1D). This effect is seen with or without other nearby cryptic /GU splice sites (Figs 1 and 4B) and can be replicated at a downstream location (Fig 4D). We believe this difference between Type III suppressors and previously identified suppressors supports the idea that these factors may act at a different point in the splicing cycle. The first U1 dependent step of 5’ss identification can be thought of like the coarse focus on a microscope, and the Type II suppressors can be thought of as mutations to factors that maintain the general region of the identified splicing target. In later steps after U1 has left, we can think of the maintenance of the 5’ss as a more “fine focus” function, perhaps related to U6 identification of the 5’ss [76] and the Type III suppressors are mutations that alter the ability of the spliceosome to maintain the fine focus of the splice site that will be used in chemistry, an effect that is consistent with the duplicated doublet switching result (Fig 4D).

PRCC(I371F) and PRCC(null) have intriguing effects on 5’ splice site choice in native introns, mostly shifting the 5’ splice site by 1nt downstream at introns beginning with GUU or 2nt downstream at introns beginning with GUGU. About 16% of *C*. *elegans* introns begin with GUU (see Methods and [77]), similar to humans, which also have about 16% of introns begin GUU (see Methods and [78]), representing the slight under enrichment for U in the third intron position. Only about 0.7% of *C*. *elegans* introns begin with GUGU, ten-fold less compared to the human transcriptome where about 6% of human introns begin with GUGU. Perhaps the under-enrichment of GUGU introns in *C*. *elegans* could be due to a vulnerability to alternative 5’ splicing at those introns.

We noticed that the introns affected by the two PRCC mutations were often long. This effect is most pronounced when we separate out those introns that are only affected by the absence of PRCC but not affected by PRCC(I371F) (Fig 5D). While the introns affected by PRCC(I371F) appear to have a similar length distribution to the wildtype *C*. *elegans* introns lengths, the introns only affected by PRCC(null) were very long, hundreds of bases longer than average introns (Fig 5F). While the average human intron is about 5400 nucleotides long [78], the most common worm intron is just 47 nucleotides. Introns beginning with GUU or GUGU are vulnerable to changes in 5’ splice site choice in the presence of both PRCC mutations, but if those introns are very long, they are only affected by the absence of PRCC, not the point mutation. This suggests a different mechanism of action for these two mutations. It has been observed that across phylogeny, intron lengths most often fall into a bimodal distribution [79,80], possibly suggesting two different mechanisms of splicing for shorter and longer introns.

**Fig 8:**
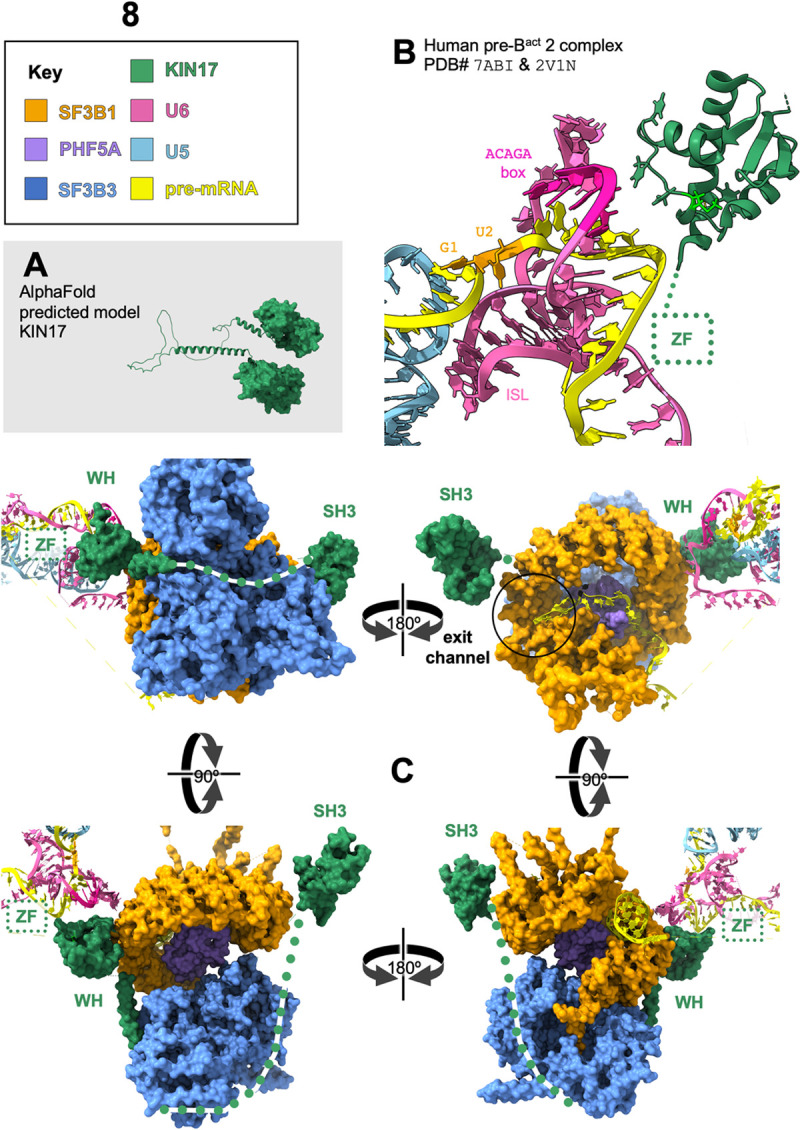
KIN17 touches 3 regions of preB^act^2 spliceosome with possible regulatory roles. **(A)** Alpha Fold predicted model of KIN17 includes two globular domains connected by alpha-helices and unstructured regions. **(B)** A model of human pre-B^act^2 complex of the splicing cycle, based on Protein Data Bank structures 7ABI [15] (positions of proteins and RNAs) with the addition of the aligned detailed structure of the loop that KIN17(M107I) resides on from PDB ID# 2V1N [25]. Colors are as noted in the key. KIN17 is near the U6/pre-mRNA helix. The pre-mRNA intron is unstructured behind KIN 17. Methionine 107 (spring green) is part of a short 3_10_ helix, on a loop between two alpha-helices of the winged-helix, and the residue points into the globular core of the winged-helix domain away from the pre-mRNA. The 23rd residue of KIN17 is not modeled in this or any structure; for zinc finger structure prediction see Fig 2B. The dashed box shows the author-proposed location for this domain, near the internal stem-loop (ISL) of U6, an important component of the eventual active site. **(C)** Pre-B^act^2 spliceosome in four orientations, colors as noted in key, with author-proposed regions shown as dotted lines. The winged-helix of KIN17 is modeled to the closed hinge of SF3b1 (HEAT repeats 15 and 16), the unmodeled zinc finger is proposed to be near the pre-mRNA U6 helix, the disordered central domain of KIN17 is proposed to loop around SF3b3, the tandem of SH3 domains is modeled on the far side of SF3b1, occluding the binding location of Prp2 (see S4 Fig for Prp2 binding in activated B^act^). In the upper right orientation, the pre-mRNA branchpoint, colored black, is encircled within SF3b1, the downstream pre-mRNA is visible exiting SF3b1 via the “exit channel” inside black circle on the upper right.

While we were preparing this manuscript, a structure of the pre-B^act^2 spliceosome was published [15], with the winged-helix of KIN17 modeled in this transient intermediate near the ACAGAGA box of U6 as it “escorts” the 5’ splice site as the spliceosome is forming the active site (Fig 8). Methionine 107 points down into the core of the globular domain, however mutations to methionine 107 could reposition nearby highly conserved aromatic residues; for example, the closest residue on the KIN17 winged helix to the U6/5’ss helix is H104, which is 5.17A from the O6 position of G46 of U6. Might this be one of those points of “fine focus”, where a nearby protein could influence the position of the pre-mRNA in the grasp of its current escort? This is the first time KIN17 has been modeled into the spliceosome, and it was found in an exciting position. Townsend *et al*., hypothesize an early transient role in spliceosome assembly for KIN17, proposing that it prevents components of the spliceosome, including PRP-8 and BRR2, from prematurely entering the B^act^ conformation. While preparing this manuscript, the AlphaFold Protein Structure Database was launched [24] allowing us to visualize the entire KIN17 polypeptide, including disordered domains which have remained elusive because they do not resolve in cryo-EM models. With this complete predicted model of KIN17 in mind (Fig 2B repeated in Fig 8A), we looked again at KIN17 modeled into the pre-B^act^2 spliceosome, this time by going into virtual reality, to see the entire structure in its 3-dimensional context [81,82]. In light of this new perspective, we take the Townsend *et al*. model a step further and propose that KIN17 might be the missing gatekeeping factor that licenses the spliceosome to proceed through assembly only after checking that the important factors are in their correct positions. Most of KIN17 is positioned in the core of the spliceosome: the zinc-finger is near what will be the active site (Fig 8B); the back of the winged-helix binds directly to the hinge of SF3b1 in the closed conformation; a long flexible linker reaches out of the core of the spliceosome; and finally on the far side of SF3b1 (Fig 8C), the tandem of SH3 domains occlude the binding site of the helicase PRP2 (S4 Fig). This occlusion of PRP2 may have implications for advancing spliceosome complex assembly, since in a later step PRP2 will pull on the downstream end of the pre-mRNA and initiate conformational changes necessary for construction of the active site. Could mutations in KIN17 be disrupting that licensing role and leading to premature PRP2 activity, selection of an upstream branch point and consequent selection of an upstream 3’ splice site? In B^act^, the pre-mRNA is held within the ring of SF3b1, the proximal pre-mRNA is in a helix with U2, the branchpoint itself is held by residues of SF3b1, and the distal pre-mRNA exits the ring to loop out of the spliceosome core structure where it will interact with PRP2 (S4 Fig) [83]. Supporting this hypothesis, there are a series of SF3b1 mutations in the “exit channel” found in human cancers which cause a shift towards the use of degenerate upstream 3’ splice sites [84].

We have demonstrated in our genetic approach that KIN17 and PRCC are splicing factors with a role in maintaining the fine focus of 5’ss splice site identity as it is loaded into the active site. As these factors appear to interact transiently with the spliceosome, our study demonstrates the importance of genetic approaches to complement the static images of spliceosome structures in order to understand the roles that these factors have in helping to guide the spliceosome during its complex rearrangement cycle.

## Methods

Full step-by-step protocols of many of the methods described below have been deposited at https://dx.doi.org/10.17504/protocols.io.p9kdr4w.

### Growth conditions

*C*. *elegans* were maintained at 20°C on nematode growth medium (NGM) agar plates inoculated with OP50 *E*. *coli*. Strains were discovered in the suppressor screen, genetically engineered using CRISPR mutagenesis, created by doing genetic crosses, or obtained from the *C*. *elegans* Gene Knockout Consortium [60].

### C. elegans strains

*C*. *elegans* strains used in this study were derived from the original Bristol N2 wild type isolate [85]. Table 2 lists the strains used, their genotypes and notes on their phenotypes.

**Table 2:** Genotypes of *C*. *elegans* strains used in this study.

Strain Name	Allele Names	Allele Descriptions
N2		wild-type isolate
SZ181	*unc-73(e936)*	/G/UU cryptic 5’ splice site uncoordinated strain
SZ283	*unc-73(e936)dxbp-1(az105)I*	Suppressor of unc-73(e936), KIN17(K23N)
SZ162	*unc-73(e936)dxbp-1(az33)I*	Suppressor of unc-73(e936), KIN17(M107I)
SZ280	*unc-73(e936)I;prcc-1(az102)IV*	Suppressor of unc-73(e936), PRCC(I371F)
SZ281	*unc-73(e936)I;prcc-1(az103)IV*	Suppressor of unc-73(e936), PRCC(Δ298–377)
SZ219	*unc-73(az63)I*	CRISPR mimic of unc-73(e936)
SZ391	*unc-73(az63)dxbp-1(az121)I;dpy-10(cn64)II*	CRISPR mimic of unc-73(e936) and dxbp-1(az105)(K23N)
SZ222	*unc-73(az63)dxbp-1(az52)I*	CRISPR mimics of unc-73(e936) and dxbp-1(az33), KIN17(M107I)
SZ308	*unc-73(e936)I;prcc-1(az122)IV*	Suppressor of unc-73(e936), CRISPR mimic PRCC(I371F)
SZ348	*unc-73(e936)I; prcc-1(gk5556)IV*	gk5556 is deletion of all coding region of prcc-1, PRCC(null)
SZ325	*dxbp-1(az137)I/hT2 I*,*III*	Deletion of KIN17(null)/HT2 over GFP balancer
SZ159	*unc-73(e936az30)I*	Intragenic suppressor of unc-73(e936) (doublet only) 2-Choice
SZ300	*unc-73(e936az30)dxbp-1(az121)I*	unc-73(e936az30) background, CRISPR mimic KIN17(K23N)
SZ224	*unc-73(e936az30)dxbp-1(az52)I*	unc-73(e936az30) background, CRISPR mimic KIN17(M107I)
SZ301	*unc-73(e936az30)I;prcc-1(az122)IV*	unc-73(e936az30) background, CRISPR mimic PRCC(I371F)
SZ263	*unc-73(az100)I*	unc-73 CRISPR-engineered reporter construct (doubled doublet)
SZ324	*unc-73(az100)dxbp-1(az121)I*	doubled doublet unc-73 with KIN17(K23N)
SZ310	*unc-73(az100)dxbp-1(az52) I*	doubled doublet unc-73 with KIN17(M107I)
SZ320	*unc-73(az100)I; prcc-1(az122)IV*	doubled doublet unc-73 with PRCC(I317F)
SZ340	*smg-4(az152)V*	CRISPR null allele of smg-4
SZ345	*unc-73(e936az30)dxbp-1(az121)I;smg-4(az152)V*	NMD mutant, CRISPR mimic KIN17(K23N)
SZ355	*unc-73(az63)dxbp-1(az52)I; smg-4(az152)V*	NMD mutant, CRISPR mimic KIN17(M107I)
SZ346	*prcc-1(az122)IV; smg-4(az152)V*	NMD mutant, CRISPR mimic PRCC(I371F)
SZ356	*prcc-1(gk5556)IV; smg-4(az152)V*	NMD mutant, PRCC(null)
VC4596	*dxbp-1(gk5666[loxP+Pmyo-2*::*GFP*::*unc-54 3’UTR + Prps-27*::*neoR*::*unc-54 3’ UTR + loxP])/+ I*.	Gene Knockout Consortium Heterozygous *dxbp-1* deletion
VC4484	*prcc-1(gk5556[loxP+myo-2p*::*GFP*::*unc-54 3’UTR + rps-27p*::*neoR*::*unc-54 3’ UTR + loxP]) IV*.	Gene Knockout Consortium homozygous *prcc-1* deletion

### Primers for *unc-73* Genomic PCR and Sequencing

Forward primer            tcaaccagaagctgttggtg

Reverse primer            tcccttaaagtaggctcgtg

### Mutagenesis and identification of putative suppressed strains

Age-synchronized uncoordinated *unc-73(e936)* hermaphrodites in gametogenesis, larval stage L4, were soaked in 0.5mM N-nitroso-N-ethyl urea (ENU) as previously described [16]. After extensive washing, four animals were placed at the edge of an OP50 *E*. *coli*-seeded 10cm NGM-agar plate, for 500 plates, and allowed to self-propagate. NGM plates were maintained at 20°. Whereas the *unc-73(e936)* animals’ movement defects confine them in place, after 8 days, suppressed F2 animals are able to crawl away from the crowded pile of uncoordinated animals, and are identifiable by their improved locomotion on the far side of the plate.

### Identification of extragenic splicing suppressors

The *unc-73* gene in suppressed lines from this screen was sequenced +/- 250bp from the *e936* mutation to distinguish between extragenic and intragenic suppressors; one of these intragenic suppressors, *unc-73(e936az30)* is used in this study (Fig 4A). Remaining extragenic suppressor alleles were mapped to chromosomes using a strategy described in [23,86]. Briefly, each suppressor strain identified in the genetic screen was crossed against a polymorphic Hawaiian isolate CB4856, and uncoordinated F2 animals that continued to have only uncoordinated offspring were recovered. These new Unc strains were then screened for regions that are homozygous for snip-SNP markers as described by [23]. Approximately 20 uncoordinated strains for each extragenic suppressor strain outcrossed to the Hawaiian strain were recovered and DNA extracted and combined. For each chromosomal region, we expected to see a mix of Hawaiian and Bristol N2 single nucleotide polymorphisms (SNPs), except in the region linked to the suppressor mutation, where we expect to see 100% Hawaiian SNPs (loss of the suppressor in the N2 background) and in the region of *unc-73* where we expect to see 100% N2 SNPs (the uncoordination allele is in the N2 background). Using this approach, we were able to narrow down the suppressors to approximately one third of the length of a chromosome. At the same time, we performed high-throughput genomic sequencing of the suppressor strains. We used STAR [87] to map those sequences back to the *C*. *elegans* genome. Diploid SNPs relative to the original N2 strain were identified using GATK [88]. The snpEff tool [89] was used to identify SNPs within genes in the chromosomal region identified by the Hawaiian strain mapping. That list of putative suppressors was cross-referenced to the Jurica lab Spliceosome database, [90], (http://spliceosomedb.ucsc.edu/) and candidate spliceosome-associated genes and RNA binding proteins in the delimited genomic region were chosen for further analysis. The suppressor allele identity was verified by *de novo* re-creation of each putative suppressor allele using CRISPR/Cas9 genome editing, and those resulting in both suppression of the movement defect and molecular changes in splicing were identified as *bona fide* suppressors.

### CRISPR/Cas9 Genome editing

Cas9 guides were chosen from the CRISPR guide track on the UCSC Genome Browser *C*. *elegans* reference assembly (WS220/ce10) [63,91,92] and crRNAs were synthesized by Integrated DNA Technologies (www.idtdna.com). Cas9 CRISPR RNA guides were assembled with a standard tracrRNA; these RNAs were heated to 95°C and incubated at room temperature to allow joining. The full guides were then incubated with Cas9 protein to allow for assembly of the CRISPR RNA complex [93]. That mix, along with a single-stranded repair guide oligonucleotide was then micro-injected into the syncytial gonad of young adult hermaphrodite animals. A *dpy-10(cn64)* co-CRISPR strategy was used to identify F1 animals showing homologous recombination CRISPR repair in their genomes [94]. Silent restriction sites were incorporated into repair design so that mutations could be easily tracked by restriction digestion of PCR products from DNA extracted from single worms. Injected animals were moved to plates in the recovery buffer [93], allowed to recover for 4 hours, and moving worms were plated individually. F1 offspring were screened for the *dpy-10(cn64)* dominant roller (Rol) co-injection marker phenotype. F1 Rol animals were plated individually, allowed to lay eggs, and then the adult was removed and checked for allele of interest by PCR followed by restriction enzyme digestion and gel electrophoresis. If an F1 worm showed the presence of a heterozygous DNA fragment matching the programmed restriction site, non-rollers in the F2 generation of that worm were screened by electrophoresis of digested PCR products. Individuals that had lost the co-injection marker but were homozygous for the allele of interest were retained and sequenced at the gene of interest to verify error-free insertion of sequences guided by the repair oligo. S1 Text contains information on specifics of the CRISPR experiments performed to generate the CRISPR-induced alleles in Table 2. crRNA sequences, the repair guide oligonucleotide sequences, the forward and reverse PCR primers for single worm PCR and the restriction enzymes used on those products to identify CRISPR-engineered genes.

### Oligonucleotides for Reverse Transcription—Polymerase Chain Reactions

The oligonucleotide sequences used in the Reverse Transcription and PCR assays to measure alternative splicing are found in S1 Text.

### RNA extraction, cDNA production, and PCR amplification

RNA from indicated strains was extracted from mixed stage or L3 populations of animals using TRIzol reagent (Invitrogen), then alcohol precipitated. Total RNA was reverse transcribed with gene-specific primers using SuperScript III (ThermoFisher) or AMV reverse transcriptase (Promega). cDNA was PCR-amplified for 25 cycles with 5’-Cy3-labelled reverse primers (IDT) and unlabeled forward primers using either Taq polymerase or Phusion high-fidelity polymerase (NEB). PCR products were separated on 40cm tall 6% polyacrylamide denaturing gels and then visualized using a Molecular Dynamics Typhoon Scanner. Band intensity quantitation was performed using ImageJ software (https://imagej.nih.gov/ij/). For quantitation, a box of the same size was drawn around each alternative splicing product on a gel in ImageJ, and a control background box of the same size was drawn between them in each lane (or just above the two if the bands were too close together). The background volume value was subtracted from each band’s value within a lane and then the relative usage of the splice sites was calculated.

### RNASeq

Triplicate total RNA isolations were done for each strain, and mRNA sequencing libraries were prepared for each RNA isolation by RealSeq Biosciences (Santa Cruz, CA). 75x75nt paired-end reads were obtained on a Novaseq 6000 sequencer, with 9 libraries combined in a lane. RNA-seq results were trimmed, subjected to quality control, and two-pass aligned to UCSC Genome Browser *C*. *elegans* reference assembly (WS220/ce10) (this earlier assembly release was used to facilitate comparison to previous RNA-seq datasets obtained by our lab) using a modified version of STAR [87]. The standard version of STAR, in addition to the canonical GU/AG intron motif, supports GC/AG and AU/AC motifs for the 5’ and 3’ splice sites. Because *C*. *elegans* does not have minor spliceosomes with AU at the 5’ end of introns, we modified the STAR source code to use UU/AG as the third motif in place of AU/AC. Furthermore, we ran STAR with parameters that adjusted the default “scoreGapATAC” (effectively scoreGapUUAG in our modified version of STAR) junction penalty from -8 to 0 so that the program would treat UU/AG spliced introns with the same scoring as GU/AG introns.

### High stringency ΔPSI analysis

Alternative 5’ (A5) and alternative 3’ (A3) splicing events found in the STAR mappings of all of the libraries were identified and filtered for those introns with at least 5 reads of support (total across all samples) and a maximum of 50 nucleotides between the alternative ends (either 5’ or 3’ respectively). In addition, alternative first exon (AF), alternative last exon (AL), skipped exon (SE), retained intron (RI), mutually exclusive exon (MX) and multiple skipped exon (MS) events were derived from the Ensembl gene predictions Archive 65 of WS220/ce10 (EnsArch65) using junctionCounts “infer pairwise events” function (https://github.com/ajw2329/junctionCounts). The percent spliced in (PSI) in each sample was derived for all of these events using junctionCounts. Pairwise differences in PSI between samples for the above events were calculated. Alternative splicing events with a minimum 15% ΔPSI were included for further consideration. Each strain had 3 biological replicates, therefore between any two strains, a total of nine pairwise comparisons were possible between each suppressor strain and the SZ340 *smg-4* comparison strain for each alternative splicing event. For each suppressor strain, only alternative splicing events that showed a change in the same direction >15% ΔPSI compared to the smg-4 control in all nine pairwise comparisons (pairSum = 9) were considered. Those events with a mean ΔPSI >20% across the 9 comparisons were included for further consideration. The reads supporting that alternative splice site choice event were then examined by eye on the UCSC Genome Browser *C*. *elegans* reference assembly (WS220/ce10) to ensure that the algorithmically flagged events looked like real examples of alternative splice site choice. S4 Table has the chromosomal location, ΔPSI measurements and notes for all alternative splicing events that fit these criteria.

### Sequencing data access

Raw mRNA sequencing data for 15 libraries in fastq format, along with.gtf files for all analyzed alternative splicing events, are available in fastq format at the NCBI Gene Expression Omnibus (GEO - https://www.ncbi.nlm.nih.gov/geo/) accession GSE178335.

DNA Sequences of raw, ENU mutagenized suppressor strains deposited at the NCBI Sample Read Archive as BioProject PRJNA778860 https://www.ncbi.nlm.nih.gov/sra/PRJNA778860

Accession numbers:

SAMN22999599, SAMN22999600, SAMN22999601, SAMN22999602,

SAMN22999603, SAMN22999604, SAMN22999605

### Staging worms for staged RNA

Mixed staged worms were bleached to isolate eggs for a rough stage synchronization. We followed "Protocol 4. Egg prep" from Wormbook: Maintenance of C. elegans (http://www.wormbook.org/chapters/www_strainmaintain/strainmaintain.html) [95].

For L3 samples, we extracted RNA 34 hours post bleaching, and for adult samples, we extracted RNA 72 hours post bleaching.

### Consensus motifs

Consensus motifs were created using WebLogo [96]; https://weblogo.berkeley.edu/logo.cgi.

### Percentage GUU and GUGU

Percentages of human and worm introns starts were calculated by extracting all known introns from the UCSC Table Browser and sorting for relevant motifs.

### Statistics

P values on all figures calculated by two-tailed student’s T-test on data with unlike variance. Values were calculated for the percent spliced in at a given splice site. Variance calculated by F-statistic. * indicates p<0.05, ** indicates p<0.005.

### Multiple sequence alignments

Multiple sequence alignments were generated using the EMBL-EBI Clustal Omega MSA webtool [97]; https://www.ebi.ac.uk/Tools/msa/clustalo/).

## Supporting information

S1 Table Quantification supporting Fig 1D. (XLSX)

S2 Table Quantification supporting Fig 4C. (PDF)

S3 Table Quantification supporting Fig 4E. (PDF)

S4 Table Detailed compendium of 5’ and 3’ splicing events supporting Table 1. (XLSX)

S5 Table Quantification supporting Fig 6C and 6D. (XLSX)

S6 Table Quantification supporting Fig 7C and 7D. (XLSX)

S7 Table Quantification supporting Fig 7E. (PDF)

S1 Text This document has the CRISPR plans for the generation of new alleles *unc-73*, *smg-4*, *dxbp-1*, and *prcc-1* for this study. It also contains the oligonucleotide sequences for reverse transcription-PCR assays used for measuring alternative splicing.
(PDF)

S1 Fig RT-PCR verification supporting that new smg-4 alleles do not degrade the NMD isoform of RPL-12. (TIF)

S2 Fig Mutations in KIN17 and PRCC(null) promote usage of 3’ splice sites with minimal consensus sequence, upstream of 3‘ canonical splice sites similar to developmentally regulated alternative 3’ splicing. (A) *C*. *elegans* 3’ splice site consensus sequence for 10,000 random wild-type introns, followed by the consensus sequence of the splice sites that were reduced in the mutant strains and then the consensus sequence of the splice sites that were promoted in the strains with mutations in KIN17(K23N), KIN17(M107I) and PRCC(null) respectively. (B) Most splice sites whose usage increases in the presence of KIN17(K23N), KIN17(M107I) and PRCC(null) are either 6 or 9 nucleotides upstream of the predominant wild-type splice site. Frequency of nucleotide shift between the splice site favored in wild type, and the splice site promoted in PRCC mutant. (C) Euler diagram shows extent of overlap between intronic events with changed 3’ splice site choice in KIN17(K23N), KIN17(M107I), and PRCC(null). (D) Euler diagram shows extent of overlap between all unique intronic events with changed 3’ splice site choice in this study, compared to the developmentally regulated 3’SS switching previously identified by our lab, in which certain introns show a shift towards usage of an alternative upstream 3’ SS in the germline, which has minimal consensus sequence aside from an AG dinucleotide at the end of the intron [64].
(TIF)

S3 Fig Tissue Enrichment Analysis of mRNA-seq. (TIF)

S4 Fig PRP2 occupies the space in B^act^ formerly occupied by the SH3 domains of KIN17. A model of human activated B^act^ complex of the splicing cycle, based on Protein Data Bank structure #5Z58 [83] in four orientations, mirroring the orientations in Fig 8C, colors as noted in key, black circle indicates the exit channel where the pre-mRNA downstream of the branchpoint leaves the SF3b1 ring. The helicase PRP2 occupies the same binding site outside SF3b1 that KIN17 occupies in Fig 8C. PRP2 is required to pull on the pre-mRNA, in a subsequent step of spliceosome rearrangement.
(TIF)
