# Supplementary material for: A genetic screen in C. elegans reveals roles for KIN17 and PRCC in maintaining 5’ splice site identity
Source: PLoS Genet. 2022 Feb 10;18(2):e1010028. doi: 10.1371/journal.pgen.1010028 (PMC8865678; doi:10.1371/journal.pgen.1010028)
Supplement: S2 Table — (PDF) [file pgen.1010028.s002.pdf]

Supplemental Table 2

Fig 4C Results	Student's T-test two-sample unequal variance			
		n	p value difference in % -1	p value difference in % wt
SZ159 unc-73(e936a30)	vs	2		
	SZ300 unc-73(e936a30)dxbp-1(K23N)	3	0.000184	0.002441
	SZ224 unc-73(e936a30)dxbp-1(M107I)	3	0.001427	0.003501
	SZ301 unc-73(e936a30)prcc-1(I371F)	3	0.000211	0.004049
