# Supplementary material for: A genetic screen in C. elegans reveals roles for KIN17 and PRCC in maintaining 5’ splice site identity
Source: PLoS Genet. 2022 Feb 10;18(2):e1010028. doi: 10.1371/journal.pgen.1010028 (PMC8865678; doi:10.1371/journal.pgen.1010028)
Supplement: S3 Table — (PDF) [file pgen.1010028.s003.pdf]

Supplemental Table 3

Fig 4E Results Student's T-test two-sample unequal variance						
		n	p value difference in % -1	p value difference in % wt	p value difference in %" -1"	p value difference in % "wt"
SZ263 unc-73(az100)	vs	3				
	SZ324 unc-73(az100)KIN-17(K23N)	2	0.039511	0.042579	0.152462	0.388011
	SZ310 unc-73(az100)KIN17(M107I)	3	0.011487	0.145628	0.100761	0.051312
	SZ320 & SZ322 unc-73(az100)PRCC-1(I371F)	7	0.029563	0.242385	0.259814	0.049619
