# Supplementary material for: A genetic screen in C. elegans reveals roles for KIN17 and PRCC in maintaining 5’ splice site identity
Source: PLoS Genet. 2022 Feb 10;18(2):e1010028. doi: 10.1371/journal.pgen.1010028 (PMC8865678; doi:10.1371/journal.pgen.1010028)
Supplement: S7 Table — (PDF) [file pgen.1010028.s007.pdf]

Supplemental Table 7

Figure 7E Results. Student's T-test two-sample unequal variance					
	**SZ340**	**SZ345**	**SZ355**	**SZ346**	**SZ356**
	smg-4 only	KIN17(K23N)	KIN17(M107I)	PRCC(I371F)	PRCC(null)
n animals	20	20	20	20	20
average	132.75	17.95	51.1	30.05	0.65
variance	1809.565789	611.1026316	2787.14737	931.7342105	0.344736842
median	139	5	41.5	23	1
vs SZ340		1.38E-11	4.48E-06	2.65E-10	2.11E-11
vs SZ345			0.01703957	0.176715658	0.005522461
vs SZ355				0.133000971	0.00041036
vs SZ346					0.000380032
