# Supplementary material for: A genetic screen in C. elegans reveals roles for KIN17 and PRCC in maintaining 5’ splice site identity
Source: PLoS Genet. 2022 Feb 10;18(2):e1010028. doi: 10.1371/journal.pgen.1010028 (PMC8865678; doi:10.1371/journal.pgen.1010028)
Supplement: S1 Text — It also contains the oligonucleotide sequences for reverse transcription-PCR assays used for measuring alternative splicing. (PDF) [file pgen.1010028.s008.pdf]

S1 Text - Suzuki et al.

A Genetic Screen in *C. elegans* Reveals Roles for KIN17 and PRCC in Maintaining 5' Splice Site Identity

CRISPR Strategies and Sequences

Suppressor mutations are bold and capitalized
Silent mutations for preventing recut or for restriction sites are capitalized or starred

unc-73

ce10::chrI:4,021,905-4,022,020

*unc-73(**e936**)*
gcagtgtgcctagaaaagtgaacgatctcattcattacaactgtttggatttgaaggatttcaag**t**tagggcttgg
actttcaattaggtataattagtgaatctgttttaagaga

Engineered mimic *unc-73*(***az63***) CRISPR repair oligo
gcagtgtgcctagaaaagtgaacgatctcattcattacaactgtttggaTCtTaaggatttcaag**T**tagggcttgg
actttcaattaggtataattagtgaatctgttttaagaga

CRISPR Guide RNA Alt-R IDT	auuugaaggauuucaaggua
Forward Primer	gcagtgtgcctagaaaagtg
Reverse Primer	gagcctactttaagggacga
Restriction Enzyme	Afl II - introduced

unc-73(az100) "doubled doublet"

Engineered mimic *unc-73*(***az100***) CRISPR repair oligo * *
ctagaaaagtgaacgatctcattcattacaactgtttggatttgaaggatttcaag**T**tagggctg**GAATTCAAGTTAGGG
CTTGGA**tagtgaatctgttttaagagaggcttaatcccaggtcttatctcttgaca

crRNA and primers same as above
Restriction Enzyme EcoR1 - introduced
Sequence changes indicated by asterisks (*) introduced the EcoR1 cut site and maintained the frame

prcc-1 (I371F)

*prcc-1(**az102**)* a → t
cattgccacaagttcaaactcaaggacaaatgtcgagaagaaagcatcaa**t**ttacgtatttggccagcttggtaagagatattccagattttatag
atttt

Engineered mimic *prcc-1*(***az122***) CRISPR repair oligo
cattgccacaagttcaaactcaaggacaaatgtcgagaagaaagcatcaa**T**ttacCtatttAgccagcttggtaagagatattccagattttata
gatttt

CRISPR Guide RNA Alt-R IDT	aaagcaucaaauuacguauu
Forward Primer	agcagcatggatgtagttgg

Reverse Primer	ggtttttgatgcaagtaaaagcctg
Restriction Enzyme	SnaBI - removed

prcc-1(null)

kind gift from Moerman Lab, *C. elegans* Deletion Mutant Consortium [60]
ce10::ChrIV:13093755-13091035

Engineered null *prcc-1(**gk5556**[loxP+Pmyo-2::GFP::unc-54 3' UTR+Prps-27::neoR::unc-54 3' UTR+loxP]) IV* CRISPR repair oligo
ttgtttatttcgtctgaaattattcgtttcttcgaagaattcttctcaaaatgggccttgtggattacgc**/**gtggagtatgaaatctgcgtaga tcaacactttttaagagcttgtgctagaaatcacataattcactta

N Terminus CRISPR Guide	aggatgggccttgtggattacgc
Forward Primer	agttccgatttctttcccgc
Reverse Primer	gagttgttgattttgtggagcg
C Terminus CRISPR Guide	cgcagatttcatactccacgagg
Forward Primer	ggacaaatgtcgagaagaaagc
Reverse Primer	cagacaatctctgcctgtcc
Flanking sequences	GTTCGACATTTCAGACAATCTCTGCCTGTC<br>GGCCCATTTTGAGAAGAATTCTTCGAAGAA
Deletion size	2661 bp (all coding regions)

dxbp-1 (K23N)

ce10::chrI:11,038,694-11,038,822

*dxbp-1(**az105**)* t → a
aaaattaattatttttaaatttttacttaaaaagtgccatacaatttcaa**a**tttgtaatcctttcgattttgttcgatttgccaagtcttttgaact tcctttttcgtgttttcccatttttaaatct

Engineered mimic *dxbp-1(**az121**)* CRISPR repair oligo
aaaattaattatttttaaatttttacttaaaaagtgccatacaatttcaa**A**tt**CTGCAG**tccttt**G**gattttgttcgatttgccaagtctttt gaacttcctttttcgtgttttcc

CRISPR Guide	gcaaatcgaacaaaatcgaa
Forward Primer (in Y52B11A.10)	ttgttccttccgcgacattc
Reverse Primer (in Exon 2)	gtgcttcggaagttaagtgg
Restriction Enzyme	PstI - introduced

dxbp-1 (M107I)

ce10::chrI:11,037,151-11,037,290

*dxbp-1(**az33**)* g → a
atcggttctgctagggcgctgacttacgttcggagccaccaatcaccaaatttcagggtcacgtgcacat**a**aactctacagtatggc actcgttgacaggcttcgtccagtatctcggatcatctggaaaatgtaaaatc

Engineered mimic *dxbp-1(**az52**)* CRISPR repair oligo
Atcggttctgctagggcgctgacttacgttcggagccaccaatcaccaaatttcagggtcacgtgcacat**A**aactcCacCgtatg gcactcgttgacaggcttcgtccagtatctcggatcatctggaaaatgtaaaatc

CRISPR Guide	tgcacatgaactctacagta
Forward Primer	aatcgagattttcgccgacg
Reverse Primer	agcaccatgaagcttgttcc
Restriction Enzyme	NlaIII - removed

dxbp-1 (null)

ce10::chrI:11,038,745-11,038,820

dxbp-1
atttaaaaatgggaaaacacgaaaaaggaagttcaaaagacttggcaaatcgaacaaaatcgaaaggattacaaaaa

Engineered *dxbp-1(**az137**)* CRISPR repair oligo
atttaaaaatgggaaaacacgaaaaaggaagttcaaaaga**TC**t**TTAA**caaatcgaacaaaatcgaaaggattacaaaa

CRISPR Guide	aaaaggaagttcaaaagacttgg
Forward Primer	tggcacatttggcagaagc
Reverse Primer	tcgcggattttcgttgttcc
Restriction Enzyme	BglII - introduced

smg-4 (null)

smg-4
tttgatgttttgcttctaaagtcataaacatttttcag/gttcgaccgatgtgcttatgcaacgcttacagtaaatttttccgaatactgtgat tcaatga

*smg-4(**az152**)* beginning of exon 2/4
tttgatgttttgcttctaaagtcataaacatttttca**A/**gttcga**A**cgatg**CAACGAACGATGCAACGAACGATGA ACGATGCGAACGATGATGACAGCAA**cttatgcaacgcttacagtaaatttttccgaatactgtgattcaatga

CRISPR Guide	taagcgttgcataagcacatcgg
Forward Primer	gaccgattcaaaagacggtc
Reverse Primer	gttctagcatcatcaggctg
Restriction Enzyme	BstBI - introduced

Oligonucleotides for Reverse Transcription - Polymerase Chain Reactions

Gene	Forward Oligo	Reverse Oligo
UNC-73	GCAGTGTGCCTAGAAAAGTG	TCGTCCCTTAAAGTAGGCTC
T21H3.9	TGTTACCGTTTCCGATGCAG	GCGAGCTTAAGAACATCAGC
M60.6	TTATCGGAAACAACGACGG	CTTGCATAATCCGCCAAACC
ATX-2	CTATCCGATGATAATGCCCC	CTATCCGATGATAATGCCCC
PANL-3	ACAACAGCCACCTACTCTGC	TTTCCCTCGAAATCGTGCCA
LMD-1	AGGTAGCCGAAATAAAACGAG	ATATCCTCGACACTGGGATC
