# Supplementary figures and images for: A genetic screen in C. elegans reveals roles for KIN17 and PRCC in maintaining 5’ splice site identity
Source: PLoS Genet. 2022 Feb 10;18(2):e1010028. doi: 10.1371/journal.pgen.1010028 (PMC8865678; doi:10.1371/journal.pgen.1010028)

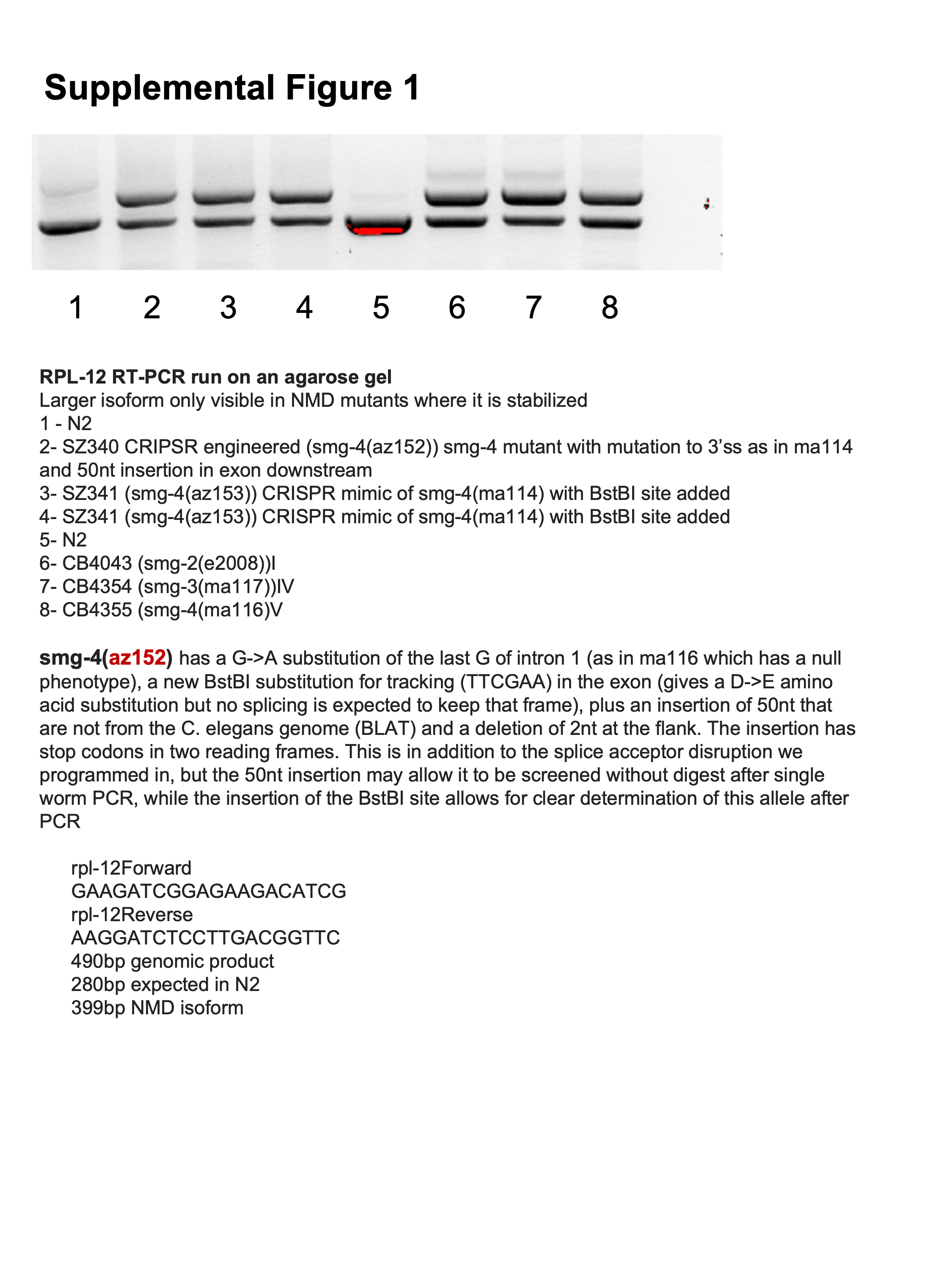

Supplement: S1 Fig — (TIF) [file pgen.1010028.s009.tif]

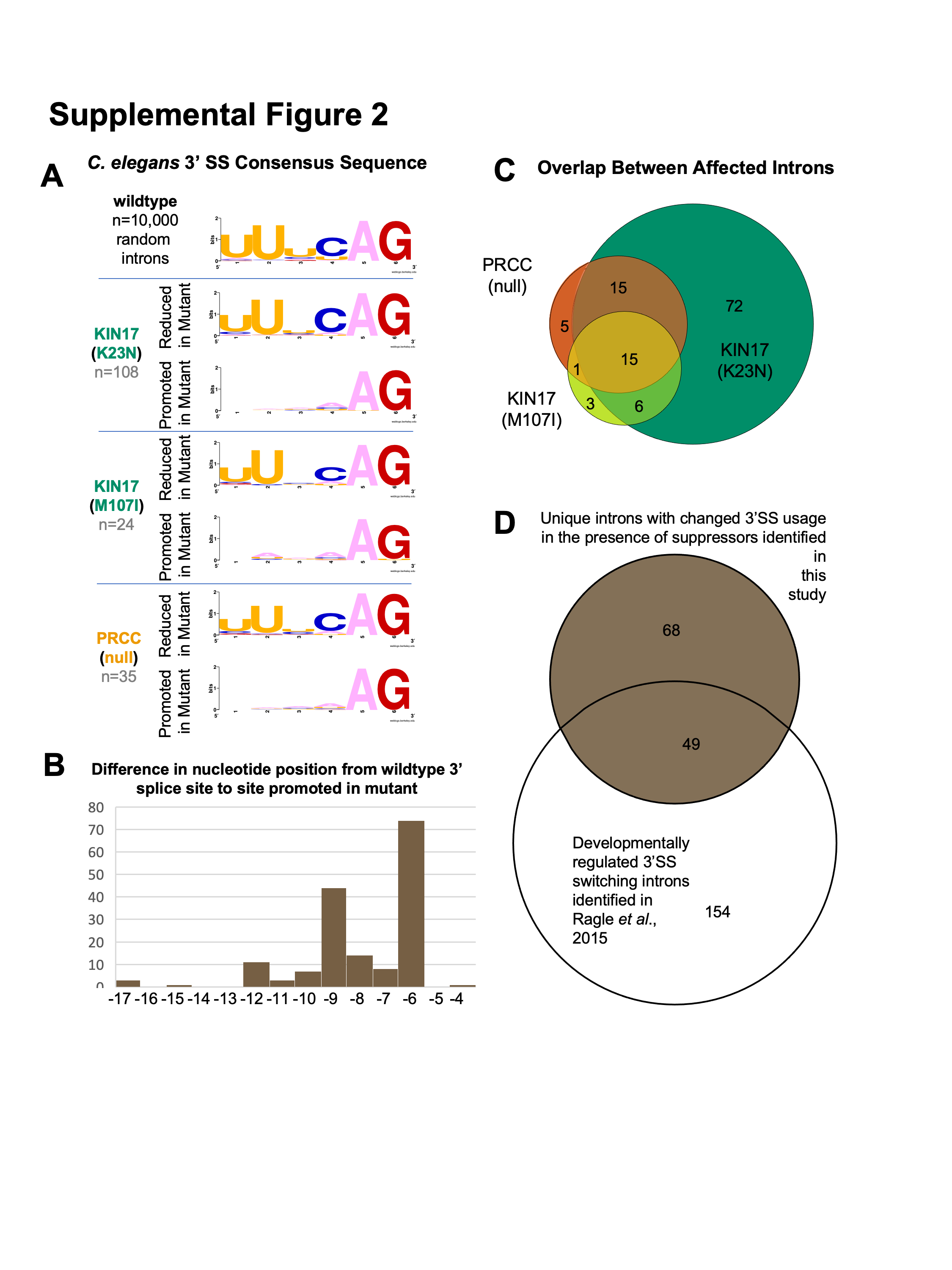

Supplement: S2 Fig — (A) C. elegans 3’ splice site consensus sequence for 10,000 random wild-type introns, followed by the consensus sequence of the splice sites that were reduced in the mutant strains and then the consensus sequence of the splice sites that were promoted in the strains with mutations in KIN17(K23N), KIN17(M107I) and PRCC(null) respectively. (B) Most splice sites whose usage increases in the presence of KIN17(K23N), KIN17(M107I) and PRCC(null) are either 6 or 9 nucleotides upstream of the predominant wild-type splice site. Frequency of nucleotide shift between the splice site favored in wild type, and the splice site promoted in PRCC mutant. (C) Euler diagram shows extent of overlap between intronic events with changed 3’ splice site choice in KIN17(K23N), KIN17(M107I), and PRCC(null). (D) Euler diagram shows extent of overlap between all unique intronic events with changed 3’ splice site choice in this study, compared to the developmentally regulated 3’SS switching previously identified by our lab, in which certain introns show a shift towards usage of an alternative upstream 3’ SS in the germline, which has minimal consensus sequence aside from an AG dinucleotide at the end of the intron [64]. (TIF) [file pgen.1010028.s010.tif]

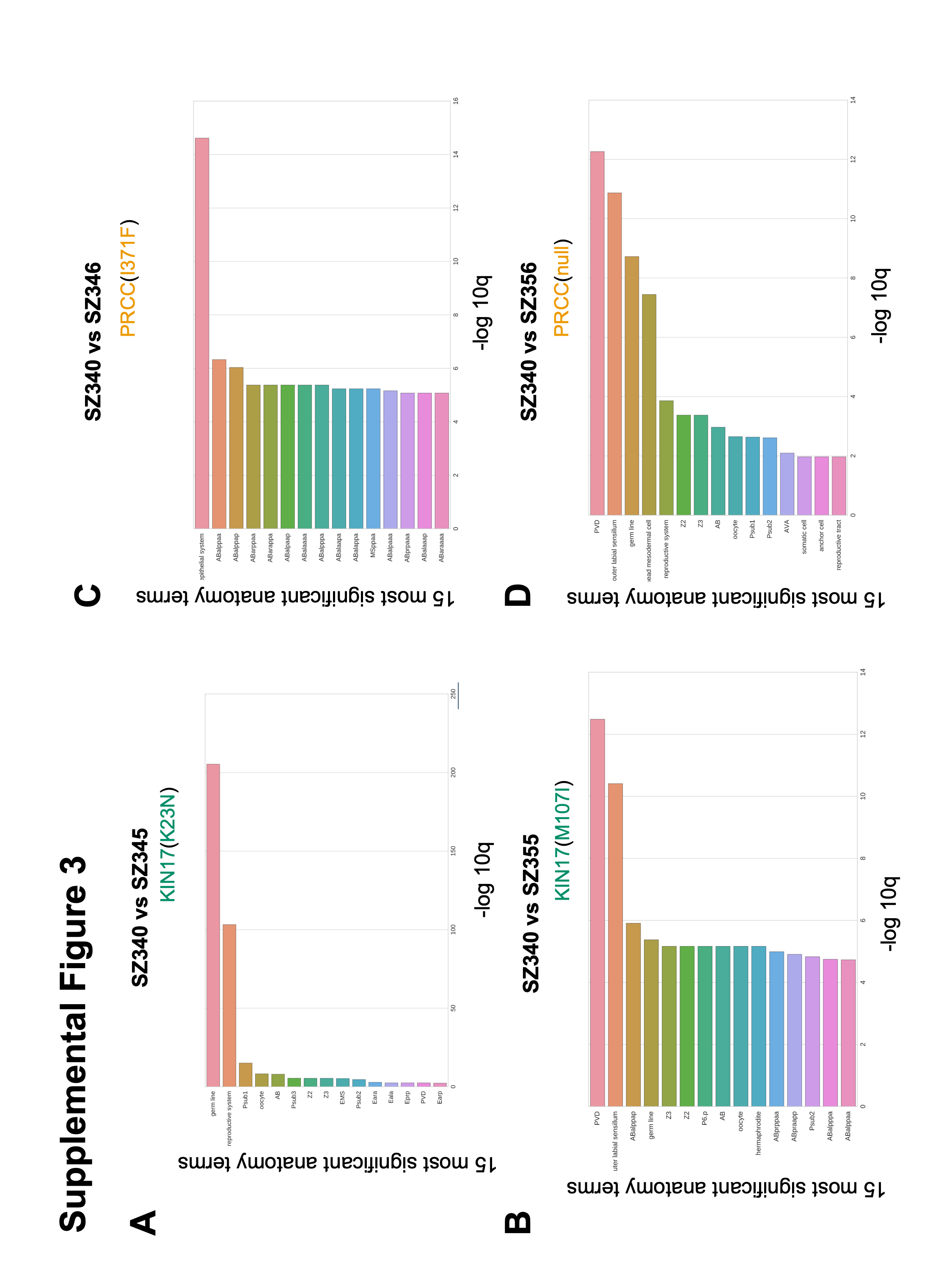

Supplement: S3 Fig — (TIF) [file pgen.1010028.s011.tif]

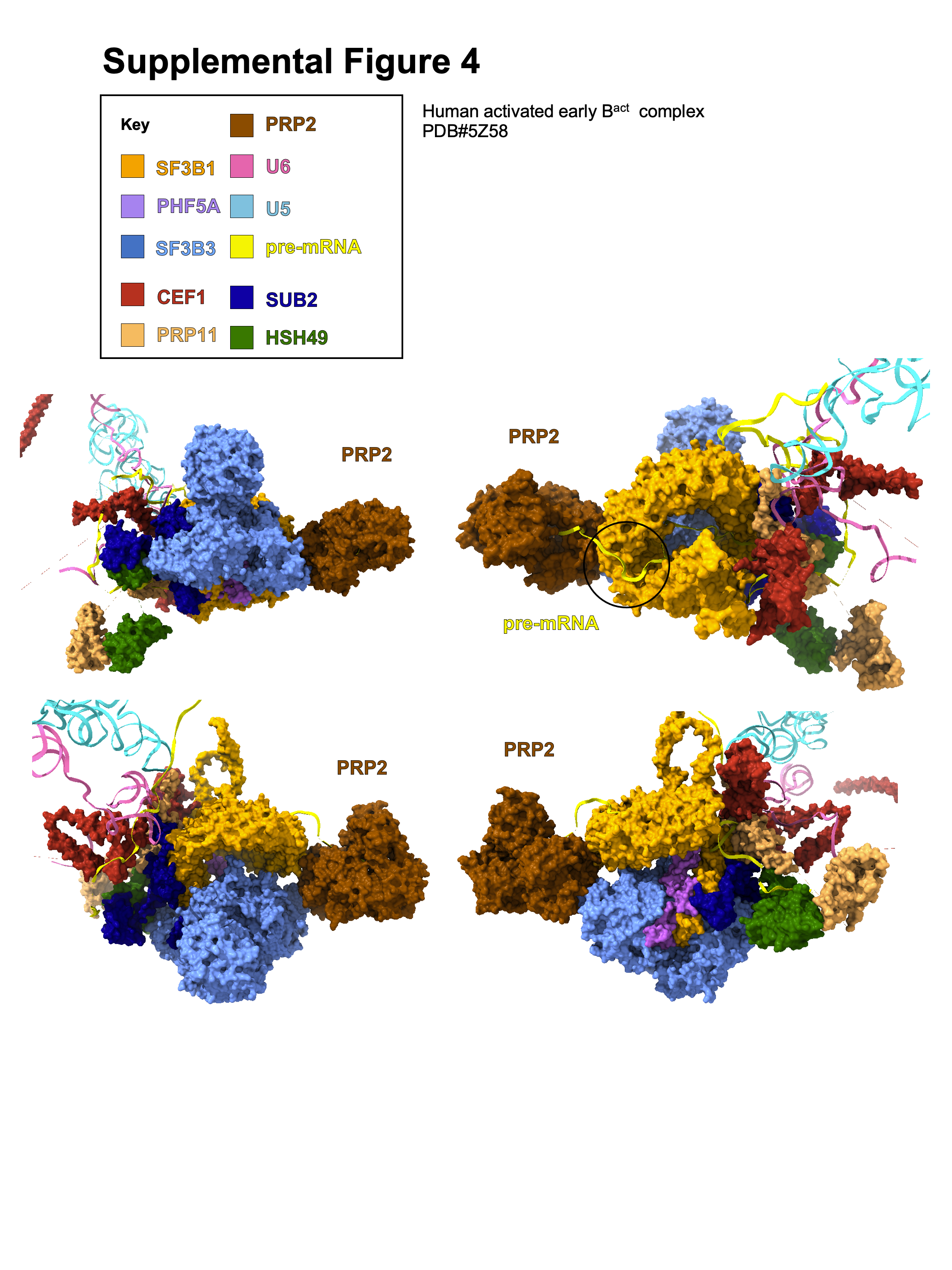

Supplement: S4 Fig — A model of human activated Bact complex of the splicing cycle, based on Protein Data Bank structure #5Z58 [83] in four orientations, mirroring the orientations in Fig 8C, colors as noted in key, black circle indicates the exit channel where the pre-mRNA downstream of the branchpoint leaves the SF3b1 ring. The helicase PRP2 occupies the same binding site outside SF3b1 that KIN17 occupies in Fig 8C. PRP2 is required to pull on the pre-mRNA, in a subsequent step of spliceosome rearrangement. (TIF) [file pgen.1010028.s012.tif]
